# Differential Functional Changes in Visual Performance during Acute Exposure to Microgravity Analogue and Their Potential Links with Spaceflight-Associated Neuro-Ocular Syndrome

**DOI:** 10.3390/diagnostics14171918

**Published:** 2024-08-30

**Authors:** Adrian Iftime, Ioana Teodora Tofolean, Victor Pintilie, Octavian Călinescu, Stefan Busnatu, Ioana Raluca Papacocea

**Affiliations:** 1 Biophysics Department, Carol Davila University of Medicine and Pharmacy, 050474 Bucharest, Romania; victor.pe.pintilie@stud.umfcd.ro (V.P.); octavian.calinescu@umfcd.ro (O.C.); 2 Ophthalmology Department, Carol Davila University of Medicine and Pharmacy, 050474 Bucharest, Romania; 3 Clinical Emergency Eye Hospital, 010464 Bucharest, Romania; 4 Department of Cardiology, University of Medicine and Pharmacy “Carol Davila”, Emergency Hospital “Bagdasar-Arseni”, 050474 Bucharest, Romania; stefan.busnatu@umfcd.ro; 5 Center for Innovation and eHealth, Carol Davila University of Medicine and Pharmacy, 010451 Bucharest, Romania; raluca.papacocea@umfcd.ro; 6 Physiology III Department, Carol Davila University of Medicine and Pharmacy, 050474 Bucharest, Romania

**Keywords:** Spaceflight-Associated Neuro-Ocular Syndrome (SANS), perimacular visual field, reaction time, microgravity analogue, head-down tilt, neuromechanics

## Abstract

Background: Spaceflight-Associated Neuro-Ocular Syndrome (SANS) is a complex pathology threatening the health of astronauts, with incompletely understood causes and no current specific functional diagnostic or screening test. We investigated the use of the differential performance of the visual system (central vs. perimacular visual function) as a candidate marker of SANS-related pathology in a ground-based microgravity analogue. Methods: We used a simple reaction time (SRT) task to visual stimuli, presented in the central and perimacular field of view, as a measure of the overall performance of the visual function, during acute settings (first 10 min) of vertical, bed rest (BR), −6°, and −15° head-down tilt (HDT) presentations in healthy participants (*n* = 8). We built dose–response models linking the gravitational component to SRT distribution parameters in the central vs. perimacular areas. Results: Acute exposure to microgravity induces detectable changes between SRT distributions in the perimacular vs. central retina (increased mean, standard deviation, and tau component of the ex-Gaussian function) in HDT compared with vertical presentation. Conclusions: Functional testing of the perimacular retina might be beneficial for the earlier detection of SANS-related ailments in addition to regular testing of the central vision. Future diagnostic tests should consider the investigation of the extra-macular areas, particularly towards the optic disc.

## 1. Introduction

Long-duration spaceflights have diverse adverse effects on the health of astronauts [1]. Among these is a series of neuro-ophthalmic ailments known as Spaceflight-Associated Neuro-Ocular Syndrome (SANS) [2,3,4,5], consisting of decreased near acuity, optic disc edema, ocular globe flattening and associated hyperopia, choroidal folds, cotton-wool spots (focal areas of ischemic retinal changes), and nerve fiber layer thickening.

The exact cause of SANS and its pathological mechanism are not clear [1,6], with several explanations proposed (which are not mutually exclusive): microgravity-related shifting of the fluids towards the cephalic extremity or impeded venous return [6], changes in the pressure gradient between intraocular pressure and intracranial pressure (the translaminar gradient) [7], supposed dysfunction of the ocular glymphatic clearance pathways [8], vigorous physical activity [5], changes in body weight [9], changes in folate- and vitamin B12-dependent 1-carbon metabolism [10], the hypercapnic environment in space stations [11,12,13], or increased hemodynamic pulsatility in the proximal-to-distal/capillary-to-venous cerebral circulation [14].

Even if the clinical manifestations of SANS appear after months of spaceflights, the changes in the retinal structures might begin very early in the flight (within the first 3 weeks), as shown by optical coherence tomography (OCT) scans performed during flights [6]. These early changes include increases in the size of the optic nerve head, choroidal folds, and an overall increase in the peripapillary total retinal thickness in majority of the astronauts. Some of these anatomical changes persist for a longer duration after flight [15]. A detailed analysis of OCT before and after flight suggested that the intracranial pressure (ICP) increase hypothesis is insufficient to explain the observed retinal changes [16].

Despite the research conducted since the discovery of SANS, we do not yet have a comprehensive set of diagnostic biomarkers or countermeasures, possible treatments, or reliable ground-based research models [17], which is a concerning problem for the short- and long-term health of astronauts.

The present pilot study aims to investigate the use of the differential performance of the visual system (central vs. perimacular visual function) as a candidate marker of SANS-like pathology in ground-based microgravity analogue conditions.

There are several analogues of microgravity that are used on the ground to simulate and investigate the effects of spaceflight on humans [18,19]. Among these, in the present study, we used bed rest (BR) [20], head-down tilt (HDT) at −6° [21,22], and HDT at −15° [23].

In previous studies conducted in microgravity analogues, various SANS-like anatomical modifications were observed [6,24,25]. Despite the documented anatomical changes, the usual visual function tests (refractometry and perimetry) do not seem to change in healthy subjects (on short HDT like 2 days [24] or even after 2 months of HDT) [26]). Despite the normal visual function tests, it has been found that HDT at −6° reduces the neurophysiological function of the retina and visual cortex (electroretinogram and visual evoked potentials) [27].

Most of the previous studies focused on physiological or structural changes that appear after longtime exposure to spaceflight. There is a lack of information about the timing of the beginning of the SANS-related functional and clinical manifestation in microgravity analogues. We focused our study on the very early onset of a microgravity analogue (the first 10 min) and studied the change in the visual analyzer performance (via a simple reaction time (SRT) task) and controlled for confounding factors. We chose the first 10 min because previous studies have shown that there are strong early cardiovascular and neurovegetative adaptive responses happening in this approximate time frame—see, for instance, published studies with a detailed analysis of the first 7.5 min [23], 10 min [14], 20 min [18,27].

The overall performance of the visual system in microgravity analogues is an ongoing research with conflicting or mixed results [28,29,30], probably due to broad topics investigated and different research methodologies [31,32]. One way to evaluate the performance of a system is to investigate its speed of response. It takes time for a person to respond to a stimulus (visual, auditory, etc.); this time is known as the “reaction time” (RT), and its determinants were thoroughly investigated in the past 150 years (for an introductory review in this fascinating topic, see [33] or an in-depth book [34], or for the complexities of its analysis, see [35,36,37,38,39,40]).

We used a simple reaction time (SRT) task to visual stimuli as a measure of the overall performance of the visual function in BR and HDT settings. The SRT task evaluates how rapidly a person can initiate a simple, pre-programmed response to a simple triggering signal (no recognition or decision required). The measured outcome is the total time from the onset of the stimulus to the performed motor response (a button click). Albeit simple, it requires the ability to detect the signal from background (with the inevitable exogenous or endogenous noise) and initiate a definite motor answer [41]. The task is repeated many times. RT was found to be altered by spaceflight [42], and we decided to investigate the changes in the RT data distribution in microgravity analogues in the context of SANS.

The main question of this exploratory pilot study is, is there an acute onset of SANS-related physiological changes after exposure to microgravity analogues? To answer it, we attempted to build dose–response models linking the gravitational component to RT distribution parameters.

## 2. Materials and Methods

The present study has a longitudinal, balanced design, in which we aimed to assess the visual reaction times of human participants, positioned in each one of four inclinations of the body, with eight visual conditions. As both SRT measurements and HDT measurements have a rather large number of confounding factors, we give below a detailed description of the experimental setup and procedures we followed in order to mitigate (or at least reduce) these factors.

### 2.1. Participants

A total of 8 volunteers (4 males, 4 females, median age: 23 years, age range: 22–46 years) completed the study, out of 9 recruited (1 participant left during the study due to personal reasons). The participants were students and staff at our university and volunteers, who were not reimbursed for their participation. Two of them participated previously in other psychophysical measurements (including RT experiments). The participants were asked to read and sign information and consent forms before participating in experiments. The study was conducted according to the guidelines of the Declaration of Helsinki and approved by the Ethics Committee of the “Carol Davila” University of Medicine and Pharmacy Bucharest, 14,877/26 May 2023. The study took place in the Space Physiology Lab at the Center for Innovation and eHealth of the “Carol Davila” University of Medicine and Pharmacy Bucharest; ophthalmological assessments took place in Clinical Emergency Eye Hospital Bucharest.

The inclusion criteria were healthy adults (>18 yrs.) of both sexes.

The exclusion criteria were as follows:

- Any current or past health issue that could be a hazard for the participants;
- Significant vision impairment defined as myopia over −6D, hypermetropia over +6D, or nystagmus, strabismus, or chromatic impairment of any type;
- Any previous infectious diseases or other diseases, even resolved in the previous 2 weeks prior to the measurements;
- Any current medication that could influence cognitive or motor performance;
- Any previous participation in any kind of clinical study in the previous 4 weeks prior to the measurements.

### 2.2. Health Assessment

The participants were screened before participation with (1) a self-assessment questionnaire for personal medical history and handedness (based on writing/mouse-using hand), (2) an in-laboratory optometric screening, and (3) an ophthalmic investigation.

The in-laboratory optometric evaluation aimed to evaluate the normality of the overall visual function and consisted in an evaluation of the visual field, with confrontation visual field testing and with an Amsler grid (monocular for both eyes), pupillary light reflex, visual acuity testing with a Sloan visual acuity chart (monocular and binocular), and color blindness of any type, which was screened with Ishihara test charts. Eye dominance was assessed with the hole-in-card test (Dolman method) and with the point-a-finger test (Porta test) [43].

An ophthalmologist investigated all the participants to screen out any possible unknown ocular problems. A comprehensive ophthalmic examination, performed with an SL-1E slit lamp (Topcon Co., Tokyo, Japan), was unremarkable for both anterior and posterior poles in all the participants. Eye fundus was examined after drug-induced mydriasis, achieved after the topical administration of 5 mg/mL tropicamide and 100 mg/mL phenylephrine hydrochloride solutions. In cases of optic nerve head with suspicious appearance (vertical cup-to-disc ratio exceeding 0.5, a discrepancy of more than 0.2 between the two eyes), optical coherence tomography (OCT, Topcon Co.) was performed. Pathological optic disc cupping is mostly suggestive for glaucoma [44], although there are other nonglaucomatous diseases that may lead to the excavation of the optic disc [45,46]. Among other quantitative parameters, OCT measures the thickness of the retinal nerve fiber layer (RNFL), the average and vertical cup-to-disc ratio, and the macular ganglion cell complex and compares them with a normative database, using the principle of interferometry. All parameters were within normal limits in all the study subjects. Intraocular pressure was measured with an NT-530P non-contact tonometer (Nidek Co., Gamagori, Japan). All the participants had normal eye pressures, with a median value of 17 mmHg (range: 13–20 mmHg). Refraction status was checked with a CRK-700 autorefractometer/keratometer (Charops Co., Anyang, Republic of Korea). To exclude any possible effect of the induced mydriasis, the reaction time measurements were performed after 5 days or more after the ophthalmic evaluation (even if the agents used were short-acting and their clinical effects disappeared in the same day).

Prior to every reaction time measurement, the blood pressure of each participant was measured with a sphygmomanometer. The chosen cutoff values were 140 mm Hg (systolic) and 80 mmHg (diastolic). None of the participants had any issue.

### 2.3. Body Positions

**Figure 1:**
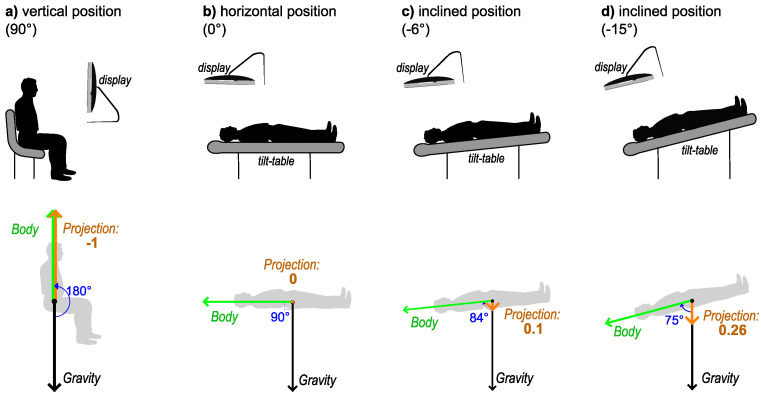
The four body positions used in this experiment. Top panels: diagrams of the arrangements of display, chair, and tilt table. Bottom panels: vector representation in the same positions (details in text).

There were a total of four possible positions for the participants (see Figure 1, top panels), as follows:

(a) Vertical position (90° angle with the horizontal plane). The participant was seated on a regular office chair and observed the visual stimuli on a monitor that was positioned so that the normal line in the center of the monitor was aligned to the midpupillary point (the middle point between the eyes). This was considered the reference position for normal daily activities. In microgravity analogue studies, positions where the head is above the horizontal line are usually called “head-up tilt” (HUT) position. Therefore, this position would be 90° HUT;
(b) Horizontal position (0° angle with the horizontal plane) on an adjustable bed (tilt table, see below);
(c) Inclined at a −6° degree angle from the horizontal (−6° HDT, “head-down tilt”, as the head is below the horizontal line);
(d) Inclined at −15° degrees from the horizontal (−15° HDT).

The above positions are named as such in the past published research on a tilt-table microgravity analogue. The terminology is very convenient as the horizontal plane is the reference plane, and it is very clear from the terminology how the body was positioned.

We propose that an equivalent description (but more advantageous in the interpretation of the data) can be given if we depict the body position as a vector representation (see Figure 1, bottom panels). The local vector of the gravity field (black arrows) is considered as the reference frame (instead of the horizontal plane). The concept of the whole body center of mass (COM), located in the lower abdomen, is useful when studying the dynamics of microgravity effects [47,48]. We define the “body vector” (green arrow in Figure 1) as the vector that starts from the COM and is directed toward the cephalic extremity. The position of the body vector is variable in the gravity field, and the relative angles between these vectors for the four positions described above are 180°, 90°, 84°, and 75°, respectively. The projection of the unit body vector on the gravity vector is proportional to the cosine of the angle between them (orange arrows and numbers in Figure 1).

For the above four positions, the relative projection values are −1, 0, 0.1, and 0.26, respectively; alternatively stated, these values represent the net value of the vertical component of the gravity force on the human body. This approach has the following benefits: it is independent of the horizontal or vertical frame of reference, it is independent of the choice of the angle measurement convention (upward or downward), and it is independent of the angle measurement system (degrees, radians). It is also easier to perform regression analysis in a physiological context as there are two extreme positions: −1 (corresponding to HUT 90°) and 1 (corresponding to HDT −90°) separated by a value of 0 (body at rest, horizontal position). The interval (−1, 0) corresponds to normal adaptation of gravity effects on the body, and the interval (0, 1) to the adverse situation of the head below the horizontal plane, where the cardiovascular system has to adapt to the blood volume redistribution.

Similar methodological approaches (to interpret the physiological data in the context of the trigonometric functions of the angle of the body) were used in previous studies [49,50,51] but often expressed the vertical component as a measure of the sine of the tilt angle (measured from the horizontal). Expressing the values in relation to the sine of the tilt angle is numerically equivalent to the cosine of the angle between the body vector and the gravity vector, but the former has the disadvantage of the requirement to always include a sign convention (“+” for HUT and “–” for HDT). The cosine transform form does not have this disadvantage; it is also commonly used in physics and vector algebra. In statistics, this trigonometric/vectorial transformation of the angular data is one of the recommended ways to effectively analyze the circular or rotational data [52,53,54,55].

### 2.4. Instrumentation

#### 2.4.1. Tilt Table

The tilt table [18,56] used in this study was a motorized, a custom-modified generic radiology examination table; the motor could raise or lower the end of the bed until its surface reached a desired inclination; and an added, embedded protractor permanently indicated the angle of the surface of the table. The tilt table had a built-in thin (~1 cm) foam cushioning over the surface of the bed area. A custom-built monitor support was rigidly affixed to the frame of the tilt table, in such a way that the monitor screen was always parallel with the surface of the bed—this ensured that the participants observed the monitor in the same relative angle to the body, regardless of the angle of the tilt table (see Figure 1b–d). The distance from the eyes of the participants to the surface of the affixed monitor was set to 60 cm. The participants were positioned in such a way that the midpupillary point was aligned with the normal line of the center of the monitor. During the measurements, the body and the head of the participants were always in direct contact with the tilt-table bed area (no pillows were used).

#### 2.4.2. Displays

The visual stimuli for reaction time measurements were presented on two identical high-performance IPS LCD (in-plane switching liquid crystal display) monitors (Dell P2419H). One monitor was attached to the tilted table (see above); the other monitor was arranged on a desk at the same distance from the eyes (60 cm) of the subject (seated on a fixed chair; see Figure 1a). One degree of visual angle corresponded to 10.47 mm on the display.

The monitors were gamma-calibrated before the experiments to ensure that the luminance/stimulus was nearly identical in both monitors: the achieved illumination difference between the displays was 0.63% (range: 0.1–1.3%) for all ranges of stimuli and positions (measured in the four quadrants of the display). The average minimum luminance (corresponding to “pure black”) was 0.69 $\mathrm{cd}/m^{2}$; the average maximum luminance (“pure white”) was 93.03 $\mathrm{cd}/m^{2}$. The Weber contrast [57] achievable by this setup was between 6.6% and 99.3%. Photometric measurements were performed with a spot photometer built in a Canon 590 IS camera. The positions of the stimuli on the monitors were arranged to fall within the best viewing angle recommended by the manufacturer of the display (within 178°). The refresh rate of the monitors was set to 60 Hz. The monitors were used after a warm-up time of at least 15 min. The monitors had a gray-to-gray response time of 5 ms, and we were careful to set all the visual items on the monitors as white, black, and shades of gray as it is known that LCD display timing accuracy is decreased for other colors [58].

#### 2.4.3. Computer Setup

For the computer setup and the presentation software, we followed the extensive recommendations for minimizing the latency described in [59,60,61,62], briefly summarized here. The monitors were connected to a laptop that generated the visual stimuli and collected responses via a digital High-Definition Multimedia Interface (HDMI) cable, to avoid electromagnetic interference or signal loss (that can happen on analogue cables). In order to avoid any cross-flickering or automatic switching of the display frequency, only one monitor was active during the measurement (also the laptop’s display was turned off). The laptop was an HP G9 ProBook model 455 running *Windows* 10 64-bit. The software used to generate visual stimuli, drive the display, and collect responses was *OpenSesame* [60] version 3.2.8, with a *PsychoPy* [63] presentation backend. The internal timings of the presentation software (for stimuli, breaks, feedback) were precomputed in “ticks” (the timing between two hardware signals driving the display [61]). In order to ensure a stable testing environment, the laptop was disconnected from the network during the entire project (to prevent inconsistent delays due to software-related interrupts [59], spurious notifications, updates, or changes in the configuration during the measurements). The collection of the reaction times was performed with an 1000 Hz button switch from a gaming mouse, designed for fast responding with minimum force and displacement (Lightsync G102, Logitech, Switzerland), ensuring a 1 ms resolution of the collected responses. On the tilt-table positions, the mouse was comfortably held in the hand, while the hand was always resting on the tilt table, around the waist level (the normal position of the hand in the supine position of the body). We were careful to ask the participants to maintain the hands in the plane with the body (no raising or lowering them) in order to avoid additional hydrostatic pressure variations due to vertical hand motion.

#### 2.4.4. Laboratory

The laboratory was artificially dimly lit (~5 lux in the measurement area), with two lamps arranged in such a way that no shadows or glare affected the monitors or the eyes of the participants. In order to exclude any possible cross-flickering effects between the lamps and monitors, the lamps and monitors had all different light intensity vs. time characteristics (monitors were LED-based with 60 Hz; one lamp was LED-based with a continuous DC current source, and the other lamp used a classic incandescent bulb with high thermal inertia—an oven bulb). Windows were shaded with opaque curtains (but the room was adequately ventilated before the measurements). The room was kept quiet during the measurements. Two authors (the same pair) were always present during the measurements, always seated on the left side of the tilt table (i.e., a consistent social environment during the measurements). The ambient temperature was kept constant (~23.5 °C) with the central heating of the building (ambient temperature can be a confounding factor for RT measurements [64]).

### 2.5. The Visual Stimuli

**Figure 2:**
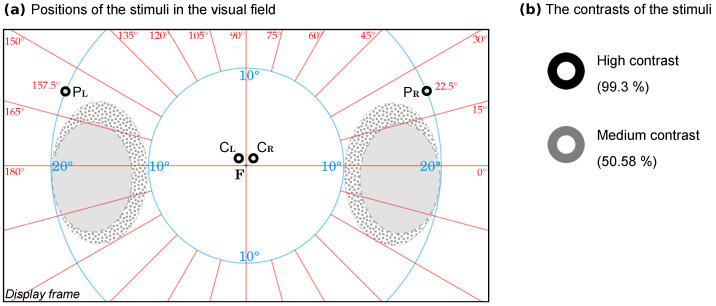
(**a**) Stimuli positions in the binocular field of view. The participants fixated the small fixation cross F on a white display. The stimulus was a 1° contrasting letter “O” presented randomly in $P_{L}$, $C_{L}$, $C_{R}$, and $P_{R}$. Gray oval shapes represent the physiological blind spots; stippled areas represent the normal variability of physiological blind spots (blind spot data are conflated from population surveys [68,69,70]). Tangent screen polar coordinates are over-imposed on this diagram as an aid to orientation: red lines represent the meridians; blue circles represent radial angles from the center (foveal fixation F). (**b**) The used Weber contrast of the stimuli (note: the values are calculated from the photometric measurement for the actual experimental display; in this diagram, the actual contrast perceived by the reader depends on the characteristics of the paper/personal display where this image is viewed).

We used a large (1° visual angle) easily recognizable visual stimulus in the shape of the letter “O” from a standard Sloan visual acuity chart, shown on a white background. We chose this annular stimulus because it is a familiar object (as opposed to gratings or Gabor patches); similar stimuli were used in the past [65]. The stimulus size was kept constant during the measurement as it is known that RT varies with stimulus size [66]. The stimulus could appear randomly only on four possible fixed locations in the visual field ($P_{L}$, $C_{L}$, $C_{R}$, $P_{R}$, in Figure 2a), while the participant fixated a small (0.25°) fixation cross F. The locations of the stimulus were as follows:

- In the central visual field, parafoveal position at a 0.5° elevation from the horizontal meridian and a 0.5° radial angle from the vertical meridian, to the left ($C_{L}$) and, respectively, to the right ($C_{R}$);
- In the perimacular visual field, at a 22.5° elevation from the horizontal meridian and at a 20° radial angle from the horizontal meridian, to the left ($P_{L}$) and, respectively, to the right ($P_{R}$).

The perimacular fields in these locations are more vascularized than the central visual field [67], being closer to the blind spot, the entry point of the major blood vessels that serve the retina. We hypothesized that, in HDT experimental paradigms (body inclination below 0°), these retinal areas would be the first affected by a change in the intracranial pressure induced by the tilt, as opposed to central areas, which are devoid of vascularization. We wanted to investigate if the change in the flow that follows the cephalad redistribution of the blood volume in HDT is immediately followed by functional changes in these areas.

We chose $P_{L}$,$P_{R}$ as these locations are as close as possible to the optic disc, adjacent to retinal artery branches (which are denser superior and inferior to the optic disc [67]), while avoiding the physiological blind spot, located at ~15° temporally for each eye, which could be a confounding factor in these measurements. Blind spot location and size vary slightly from person to person; the size is also dependent on the stimulus size (smaller stimuli used for testing yield a larger blind spot area) [68]. As we presented the stimuli on a flat computer screen, we were careful to avoid the spherical projection distortion of the blind spot location on its flat surface—as locations are reported in the literature in polar coordinates obtained either with spherical Goldman-type perimeters or with a flat tangent screen method. We conflated the data from several studies that reported detailed information of the blind spot location and its normal variance using flat projection [68,69,70]; see the gray ovals (blind spot) and the stippled areas (variations) in Figure 2a).

It is known that the reaction time can be influenced by prior experience [71]; we attempted to reduce this confounding factor by introducing a slight variation of the appearance of the visual stimuli. Half of the time, the presented stimuli were black (photometric-measured Weber contrast: 99.3%), and the other half of the stimuli were dark gray (contrast: 50.58%) (see Figure 2b). This latter value is well above ~0.5%, which is the contrast threshold for this type or target [57], so it is not posing any challenge in recognition. We carefully considered the choice of a darker stimulus against a white background, as this combination is known to minimize SRT latency [59]; SRT latencies are influenced by contrast but more importantly by overall brightness (total luminous energy) [72,73,74]. The order of presentation was fully randomized in each measurement.

### 2.6. The Experimental Protocol for Each Participant

In order to reduce the practice effects on reaction time for central vs. peripheral fields (a confounding factor, [65]) we split the measurement sessions for each participant in different days, with a break of 10–28 days between them. For each participant, we followed this timeline:

- In the initial presentation day in the laboratory, we explained the procedure and showed a short demonstration of the measurement equipment, obtained consent, asked for the self-assessment questionnaire for personal medical history and handedness, and performed an in-laboratory optometric screening, followed by a break (~10 min);
- Two measurements (in two different body positions) in random order. Between them, there was a break of at least 30 min when the participant rested vertically (on a chair or walked for a break);
- Followed by a break of 5–14 days (depending on the schedule of the participants);
- Followed by the comprehensive ophthalmic investigation (in the hospital);
- Followed by a break of 5–14 days (depending on the schedule of the participants);
- Followed by a day in the laboratory when the last two measurements were performed (in two different body positions), in random order, with a break of 30 min (as in the first day).

For the participants, the order of the measurements in each one of the four body positions was randomized with a block rotation design.

### 2.7. The Measurement Session for Each Participant

As the intention was to study the initial impact of the onset of the microgravity analogue on the performance, we set the first 10 min spent on each body position (tilted table or chair) as a time limit for our study. This 10 min was split as follows:

- The participants seated themselves on the tilted table (or chair);
- A 2 min initial adaptation time: the table was adjusted to the desired angle, and then we waited for a maximum of 2 min to check the stability of the position of the participants, to check for any issues reported by the participants (if any), and to perform a quick verification of the response time experiment (8 practice trials, which were discarded and not included in the analysis);
- A 6.2–8 min time to perform the reaction time measurement (SRT trials, see below);
- Coming back to the initial position.

In this narrow time frame, we could fit at most 112 SRT trials in total.

For each discrete condition in this narrow time frame, we could fit 14 SRT trials (above the minimum range of 5–10 trials recommended by [75]). Therefore, for each body position, for each participant, we collected a total of 112 response times (in the range of 100 trials recommended by [40] for minimizing the errors of fitting). During the measurements, the participants could take a short break (if they wished so) every 20 trials. During the break, they were asked to remain in the same position.

#### 2.7.1. The Timeline of a Measurement

**Figure 3:**
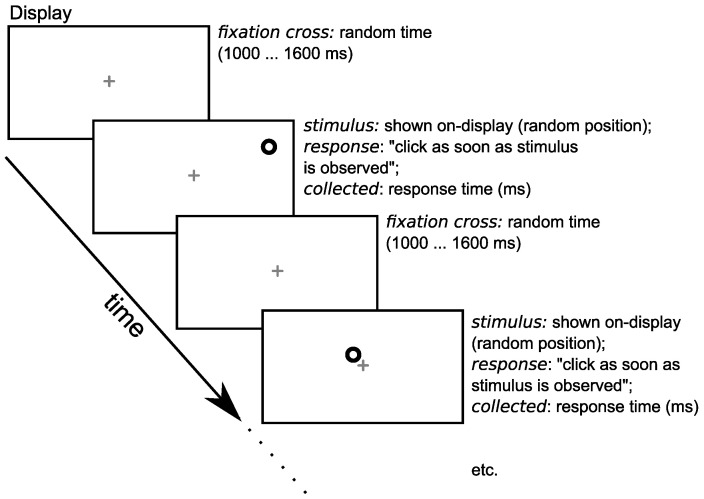
The timeline of a measurement in each body position. A measurement consisted of a sequence of SRT trials. Two example trials are depicted; a trial consisted of fixation screen, followed by a stimulus screen.

A measurement consisted of a sequence of SRT trials. In each trial, the participants were asked to continuously maintain their gaze on a small gray fixation cross (0.25 degrees) in the center of the visual field (Figure 3). After a random delay (1000 to 1600 ms), the visual stimulus appeared randomly on one of the preset locations ($P_{L}$, $C_{L}$, $C_{R}$, and $P_{R}$) and with a random contrast (Figure 2b). The task of the subject was to react as quickly as possible to the appearance of the stimulus, via a button press on the switch (using the index finger of the dominant hand). The stimulus remained on the display until the participant responded. To maintain the attention, auditory feedback was provided (a short beep, 150 ms, 880 Hz) after each response, as it is known from previous research that feedback is advisable when the speed of the response is the main interest of the study [41]. The computer setup collected the response times between the display of the stimulus and the key press. After the key press, a new trial was started and so on. About every 25 trials, the participant could take a small break if desired (but remaining seated in the same position). The order of stimulus positions and contrast were varied in a completely randomized block design in order to reduce the carry-over effects. The random delay time was sampled from a uniform distribution.

#### 2.7.2. Time

In order to avoid confounding effects from seasonal changes or physiological circadian rhythm [76], all reaction time measurements were scheduled at the same time of day (afternoon, 14:00 to 17:00). The measurements were performed during the winter of 2023–2024.

### 2.8. Statistics

#### 2.8.1. At the Participant Level

The variable collected was the simple reaction time (RT) to each visual stimulus presented, in milliseconds. We collected a total of 3584 RTs from the eight participants (448 RTs per participant). For each participant, for each experimental condition, we calculated the following parameters: *the mean of RT, RT standard deviation (Std.Dev.), median RT, skewness, and kurtosis.*

The use of the means reflects the implicit assumption of a Gaussian distribution, but it is known that RTs are not normally distributed [34,36]. Therefore, instead of normalization of the raw RT data (via the elimination of outliers, nonlinear transformations of the measurement scale, etc.), we preferred to additionally analyze the full distribution of the RT data. It is known that the analysis of the distribution brings more information about the underlying processes [35,36,38,39,40,77]. Among the several distributions used, we chose the ex-Gaussian distribution for its simplicity and because it fits RT data very well [35,37,40]. Briefly, the ex-Gaussian distribution is a right-skewed curve, described by three parameters: μ, σ, and τ. It is a convolution of two well-known distributions: a Gaussian distribution (mean μ and standard deviation σ) and an exponential distribution (with the rate parameter τ). Visually, the right slope is related to the μ parameter, the breadth to the σ, and the left slope to the τ parameter. See [78] for a graphical overview of the useful distribution and [38] for a detailed informative description and comparison with other distributions used in RT research.

For each condition and participant, we fitted an ex-Gaussian distribution on the measured RT data, using a dedicated package for fitting this distribution [79], with a bootstrapping method with a large number (50.000) of iterations per fit. For each modeling procedure, in order to compare the goodness of fit, we calculated the following indicators: Akaike information criterion (AIC), Bayesian information criterion (BIC), and log-likelihood estimate (LogLIK).

#### 2.8.2. At the Group Level

We looked at the above parameters calculated at the individual level, and analyzed them at a group level, for all the participants. The normality of the distribution of these parameters was evaluated with the Shapiro–Wilk normality test and by visual inspection of the quantile–quantile plots. If the parameters were normally distributed, we used parametric tests; otherwise, non-parametric tests.

It is known that this groping technique can also be used for the parameters of the fitted ex-Gaussian distributions (μ, σ, and τ), since, for instance, the averaging of these parameters outperforms other methods (like quantile averaging) while forming group-level response time distributions [80].

We used linear mixed-effects modeling (LME) [81] to evaluate the impact of increasing tilt on the RT. We modeled the *body position* (as a vector projection in the gravity field) as the *fixed* effect (i.e., the condition of the participant) and the *individual participant* (ID) as a *random effect*.

We hypothesized that since there are two physiological states (HUT and HDT) separated at 0°, a piece-wise modeling (yielding two slopes) seems to be more appropriate than a single linear relationship. We therefore *a priori* set up the knot (the separation point) at a position of 0°; this will yield two slopes, one for HUT and one for HDT. In this paper, we will denote the slope for HUT as $\beta_{1}$ and for HDT as $\beta_{2}$. The advantage of this modeling approach is that there is no significant difference between the effects of the two states (HUT and HDT); the corresponding β coefficients will be similar (a co-linearity effect). Conversely, if there is a difference in slopes, this should be visible as a trend change in the models, around a position of 0°.

We took a rather conservative approach in LME modeling [82], and as such, we kept the models as simple as possible (as few parameters as possible); we compared diverse models using AIC, BIC, and LogLIK measures; and we reported the full model parameters and tests performed (in Appendix A, in order not to clutter the main text). To aid understanding, we depicted the models graphically and reported $R^{2}$. For mixed-effects models, $R^{2}$ can be categorized loosely into two types: marginal $R^{2}$ and conditional $R^{2}$. The marginal $R^{2}$ is concerned with variance explained by the fixed factors of the model, and conditional $R^{2}$ is concerned with variance explained by both fixed and random factors (i.e., by the whole model) [83]. For brevity, in this paper, we will note the marginal as $R_{m}^{2}$ and the conditional as $R_{c}^{2}$. LME models were calculated with *lme4* [84], using the same default settings proposed by its authors, for all the models reported in this paper: the fitting was performed with restricted maximum likelihood (REML) option, using “nloptwrap” (nonlinear optimization) settings. Additionally, in order to facilitate comparisons of the models reported here with future models, we calculated standardized parameters (standardized beta); these were obtained by fitting each model on a standardized version of the dataset. Using a Wald t-distribution approximation, 95% confidence intervals (CIs) and *p*-values were computed. The R language formula expression for each model followed this form: *Parameter ~ bs(BodyPosition, knots = c(0), degree = 1) + (1|ID)*, where *bs() is* the function call for the basis-spline method for polynomial splines [85], and each model included the individual participants (ID) as a single random effect (formula: ~(1|*ID*). By *Parameter*, we refer to each one of the parameters described in the above section. We only report here the statistically significant models with a noticeable effect size. In order to aid future comparisons with the present findings, standardized beta parameters of the models (Std. beta) were obtained by fitting the models on a standardized version of the dataset and reported alongside the main models (in Appendix A). Using a Wald t-distribution approximation, 95% confidence intervals (CIs) and *p*-values were computed.

For each one of the reported models, we also included the relevance of the findings with statistical tests of significance and effect size calculation (Hedges’ *g*, Kendall’s *W*, and partial eta squared, depending on the test). The effect sizes were qualitatively judged as “very small”, “small”, “medium”, and “large” according to recommendations summarized in [86,87]. We provided a graphical overview of the models in the *Results* section and a detailed description of each model parameter in Appendix A. The cutoff for statistical significance was set at 5%. In cases of multiple pairwise comparisons, the reported values are Holm-adjusted [88,89]. However, in Appendix B, we report some exploratory findings that we considered worthwhile for spotting trends, where we relaxed the p-adjustment methods as recommended in [89,90,91].

#### 2.8.3. Software

Data analysis was performed with *R* [85] version 4.3.3, with the following additional packages: *retimes* (v. 0.7.2) [79], *lme4* (v. 1.1-35-3) [84], *splines* (v. 4.4.1) [85], *ggeffects* (v. 1.7.0) [92], *emmeans* (v. 1.10.1) [93], *effectsize* (v. 0.8.9) [87], *pander* (v. 0.6.5) [94], *report* (v. 0.5.9) [95], *Polychrome* (v. 1.5.1) [96], *rstatix* (v. 0.7.2) [97], *stargazer* (v. 5.2.3) [98], *ggplot2* (v. 3.5) [99], and *tidyverse* (v. 2.0) [100]. Stimulus generation and data collection were performed with *OpenSesame* (v. 3.2.8) [60] for *Windows* 10, 64-bit version.

## 3. Results

### 3.1. Quality of the Measurements

**Figure 4:**
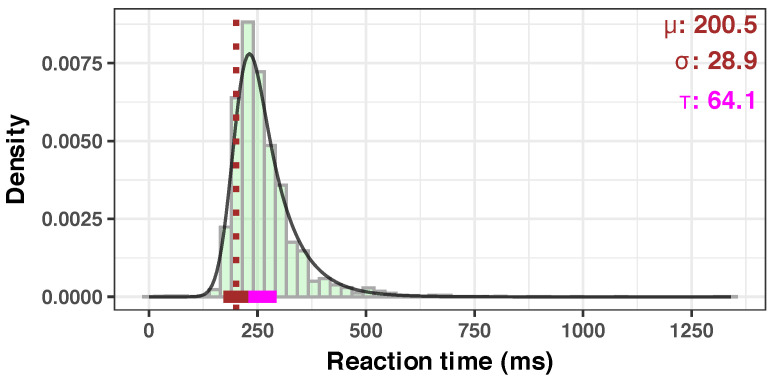
Histogram of consolidated data from all measurements; bin width is 25 ms. Black curve: fitted ex-Gaussian distribution. To visually guide the eye, the parameters of the distribution are shown: μ as a vertical brown dotted line, σ as a thick brown line, and τ as a thick magenta line. Their numerical values are shown in the top-right corner. Vertical axis: probability density (probability per unit of time; the total area under the curve is one; the total histogram area is one).

In our SRT paradigm study, the overall median response time was 248 ms, with an average of 264.6 ms (standard deviation, 81.9; range, 0.03 ms–1340.66 ms) and a 3.48 skewness. A consolidated overview of all the measured RTs shows the expected single-peaked, right-skewed distribution (see the histogram in Figure 4). An ex-Gaussian distribution that fits the overall data, shown as a black curve in Figure 4 follows the expected right-skewed pattern. We present below our findings about the impact of the body position on different parameters of RTs and their implications.

### 3.2. Overall Group Results

*Paradigm: “RT to any kind of stimulus in the visual field, in different body positions*”.

*Hypothesis*: “*An acute exposure to a microgravity analogue would alter the distributions of the visual RT*”.

**Figure 5:**
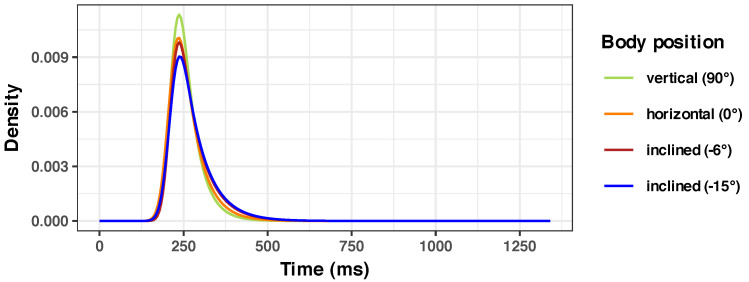
The fitted ex-Gaussian distributions calculated from the averaged parameters of each participant, in the four body positions; the numerical parameters are listed in Table A1. Vertical axis: probability density (probability per unit of time; the total area under each curve is one).

The grouping variable was the *body position*, with four levels: vertical (90°), horizontal (0° tilt), inclined at −6°, and inclined at −15°. In each one of the four body positions, there were 112 reaction times (trials) per participant. For each participant, in each position, we calculated the descriptive statistics of the reaction times: the mean, median, and standard deviation of the mean; we calculated the parameters of the ex-Gaussian distribution that best fitted the overall reaction times of each participant (mu, sigma, and tau) in each body position and the indicators of the goodness of fit (AIC, BIC, LogLIK). For each body position (vertical 90°, horizontal 0°, inclined −6°, and inclined −15°), we calculated the group averages of the above-mentioned parameters. The results are summarized in Figure 5 and are presented in detail in Appendix A.2, Table A1.

In our sample, we observed a slight but consistent increase in mean RT with 8.71 ms, from 255.09 ms (at 90° position) to 255.55 ms (at −15° tilt), and a more consistent increase in Std.Dev. with 23.8 ms (from 52.79 to 76.59 ms). There is also a gradual change in the shape of the RT distribution, as the body inclination changes from 90° to 0° (see Figure 5); a prolongation of the left slope (tau component, from a value of 40.07 at 90° to 60.22 at −15°) and reduction of the peak (or a change in kurtosis) are even visible to the eye. This seems to indicate that the change in the body position is subtly reflected in the change of the distribution of SRT to visual stimuli.

**Figure 6:**
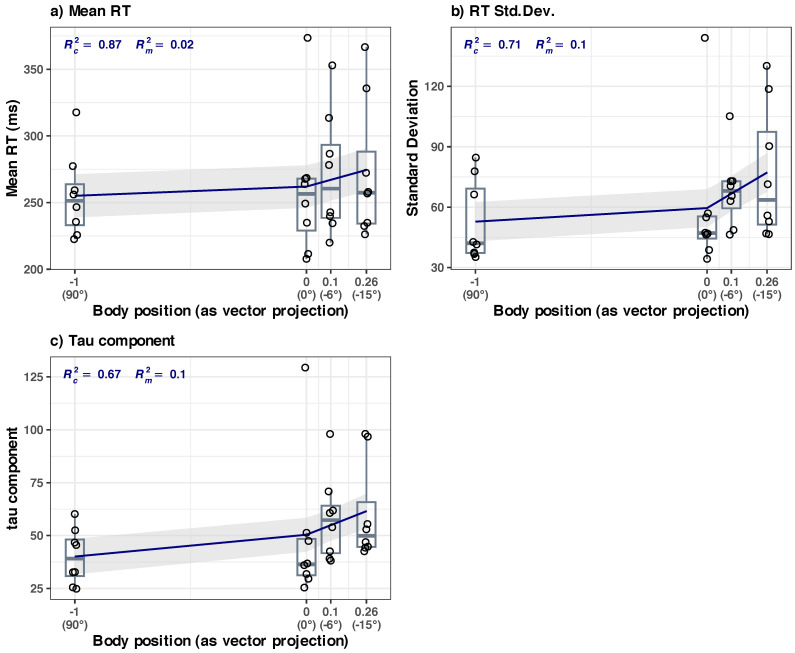
Body position influence on parameters of reaction times to visual stimuli (overall, all stimuli). LME modeling of (**a**) mean RT, (**b**) standard deviation of RT, and (**c**) ex-Gaussian fitting tau component of RT vs. body position. The body position is represented as vector projection values on the gravitational field; the corresponding degrees of body position in relationship with horizontal is shown by the numbers in parentheses. The dark blue line is the LME model on the data; the part for HUT is the line between 90° and 0°, and the part for HDT is between 0° and −15°. The gray bands are the 95% CI. The circles represent the individual values from the participants, slightly automatically jittered on the horizontal axis to distinguish overlapping values. A boxplot is shown for each position to grasp the distribution pattern at the position level. The numerical values are presented in Table A2.

Interestingly, it seems that these increases are not evenly distributed over the circular ranges of the body positions. We performed a thorough analysis of each component per condition; the significant trends observed in our sample were the correlation of the (a) mean RT, (b) RT standard deviation, and (c) tau component with the body position as revealed by a linear mixed-effects modeling (Figure 6). The full details of these three models are given in Appendix B.2 (see Table A2).

**(a) Mean RT at the group level** (Figure 6a) The LME model of the mean RT as a function of the body position has the following R formula: *Mean ~ bs(BodyPosition, knots = c(0), degree = 1) + (1|ID)*. The model included ID as a random effect (formula: ~1|ID).

The model’s total explanatory power is substantial (conditional $R^{2}$ = 0.87), and the part related to the fixed effects alone (marginal $R^{2}$) is 0.02. The model’s intercept, corresponding to body position = 0 (at 0° tilt), is at 255.09 (95% CI [221.91, 288.28], t(27) = 15.77, *p* < 0.001). Within this model are the following:

- The effect of body position [HUT] is statistically non-significant and positive ($\beta_{1}$ = 6.91, 95% CI [−8.94, 22.76], t(27) = 0.89, *p* = 0.379; Std. beta = −0.02, 95% CI [−0.53, 0.50]).
- The effect of Body Position [HDT] is statistically significant and positive ($\beta_{2}$ = 19.40, 95% CI [3.00, 35.79], t(27) = 2.43, *p* = 0.022; Std. beta = 0.44, 95% CI [0.07, 0.81]).

**Figure A1:**
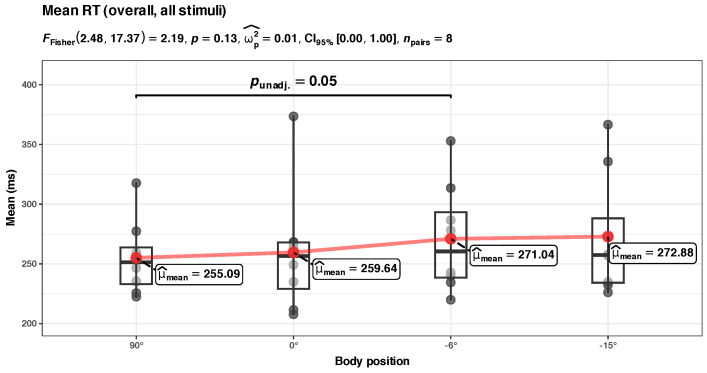
A p-unadjusted ANOVA analysis of the mean values of RT data, presented in the LME model in Figure 6a. The upward trend is visible but masked by the high variability of the data. Gray dots: mean RT values from the participants, overimposed on the boxplots of the data distribution. Red dots: the means at the group level. The red line is drawn as a guide to the eye.

*Summary*: the body position in HUT (90° to 0°) does not appear to significantly influence the mean RT. The body position in HDT significantly influences the mean RT, but the effect size is very small (in absolute terms, only about a ~7% increase in average RT when the participants went from a vertical position (90°) to an inclined (−15°) position). A regular p-adjusted repeated measures ANOVA analysis does not reveal the upward trend, but it can be spotted without the p-adjustment (see Appendix B.2, Figure A1).

**(b) RT standard deviation at the group level** (Figure 6b)

The LME model of the standard deviation of RT data has the following R formula: *StDev ~ bs(BodyPosition, knots = c(0), degree = 1) + (1|ID)*. The model included ID as a random effect (formula: ~1|ID).

The model’s total explanatory power is substantial (conditional $R^{2}$ = 0.71), and the part related to the fixed effects alone (marginal $R^{2}$) is 0.10. The model’s intercept, corresponding to body position = 0 (at 0° tilt), is at 52.79 (95% CI [32.78, 72.79], t(27) = 5.41, *p* < 0.001). Within this model are the following:

- The effect of body position [HUT] is statistically non-significant and positive ($\beta_{1}$ = 6.75, 95% CI [−8.35, 21.85], t(27) = 0.92, *p* = 0.367; Std. beta = −0.15, 95% CI [−0.92, 0.63]).
- The effect of body position [HDT] is statistically significant and positive ($\beta_{1}$ = 24.39, 95% CI [8.77, 40.01], t(27) = 3.20, *p* = 0.003; Std. beta = 0.87, 95% CI [0.31, 1.43]).

**Figure 7:**
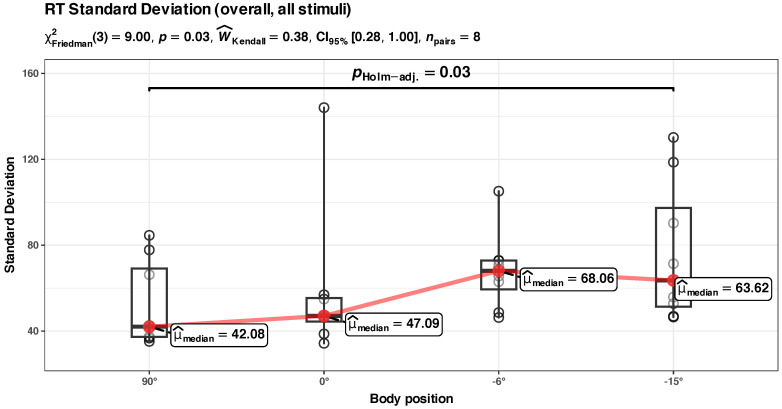
Group analysis (Friedman rank sum test) of the LME model in Figure 6b. Gray dots: calculated standard deviation of RT values from each participant, overimposed on the boxplots of the data distribution. Red dots: the sample medians at the group level. The red line is drawn as a guide to the eye. The horizontal bar shows the significant post hoc inter-group comparisons (Durbin–Conover test); *p*-values reported are Holm-adjusted. $\hat{W}$, Kendall’s coefficient of concordance. CI, confidence intervals. The difference is more obvious in the unadjusted analysis (Appendix B.2, Figure A2).

We also checked the results with a simple Friedman rank sum test (the standard deviation values in position 0° were not normally distributed), and the results seem to be in agreement (there is a statistically significant difference between the groups, *p* = 0.03, with a calculated effect size (Kendall’s $\hat{W}$ = 0.38) rated as “moderated agreement”; see Figure 7).

*Summary*: the body position in HUT (90° to 0°) does not appear to significantly influence the standard deviation of RT. The body position in HDT (0° to −15°) significantly increases the standard deviation of the RT data, and the effect size is appreciable (in absolute terms, about a ~45% increase in Std. Dev. of RT when the participants went from a vertical position (90°) to an inclined (−15°) position).

**(c) Tau parameter at the group level** (Figure 6c)

R formula: *tau ~ bs(BodyPosition, knots = c(0), degree = 1) + (1|ID)*. The model included ID as a random effect (formula: ~1|ID).

The model’s total explanatory power is substantial (conditional $R^{2}$ = 0.67), and the part related to the fixed effects alone (marginal $R^{2}$) is 0.10. The model’s intercept, corresponding to body position = 0 (at 0° tilt), is at 40.07 (95% CI [22.87, 57.28], t(27) = 4.78, *p* < 0.001). Within this model are the following:

- The effect of body position in HUT is statistically non-significant and positive ($\beta_{1}$ = 10.31, 95% CI [−3.60, 24.22], t(27) = 1.52, *p* = 0.140; Std. beta = 0.14, 95% CI [−0.69, 0.98]).
- The effect of body position in HDT is statistically significant and positive ($\beta_{2}$ = 21.46, 95% CI [7.07, 35.85], t(27) = 3.06, *p* = 0.005; Std. beta = 0.89, 95% CI [0.29, 1.49]).

**Figure A3:**
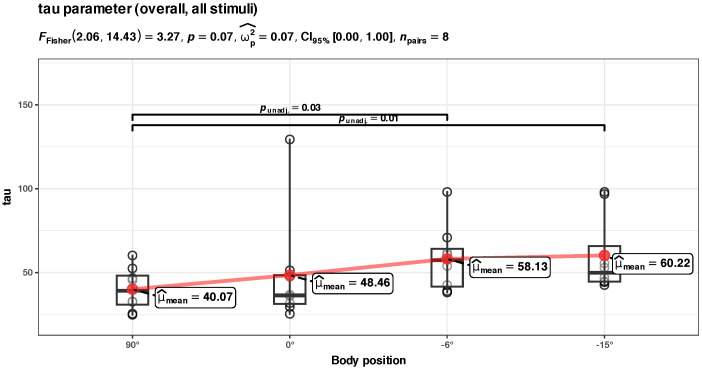
A p-unadjusted group analysis (repeated one-way ANOVA) of the data presented in LME model in Figure 6c. Gray dots: calculated tau values of each ex-Gaussian fit on RT values from each participant, overimposed on the boxplots of the data distribution. Red dots: the means at the group level. The red line is drawn as a guide to the eye. Inter-group unadjusted comparisons (Student’s *t*-test) suggest that there is a resizable difference also between 90° (vertical) and −6° and between 90° and −15°. This upward trend is picked up by the LME model, as shown in Figure 6c, even if this ANOVA analysis is not significant.

*Summary*: The body position in HDT significantly influences the tau component of the RT, the effect size is appreciable (in absolute terms, about a ~50% increase in the average tau parameter of the RT when the participants went from a vertical position (90°) to an inclined (−15°) position). There is a high dispersion of the data, a regular p-adjusted ANOVA analysis does not reveal the significant differences, but it can be spotted without the p-adjustment (see Appendix B.2, Figure A3). The body position in HUT (90° to 0°) does not appear to significantly influence the tau component of RT.

The above three models presented in Figure 6 represent the situation of the performance changes in the overall visual field, with the data collected from the four points (PL, CL, CR, and PR; see Figure 2). However, these data are a conflation of two distinct areas: perimacular (PL, PR) and central (CL, CR) fields of view. If there is an equal contribution of both areas to the performance changes, a split analysis should yield symmetrically split curves (one for perimacular, one for central). If the areas have a different contribution, an asymmetrical distribution of the split models should emerge. The next section explores this question.

### 3.3. Central vs. Perimacular Field of View

*Paradigm: “Any kind of stimuli appearing in central vs. perimacular visual fields*, *in different body positions*”.

*Hypothesis*: “*An acute exposure to a microgravity analogue would alter the distributions of the visual RTs in the central vs. the perimacular visual field*”.

**Figure 8:**
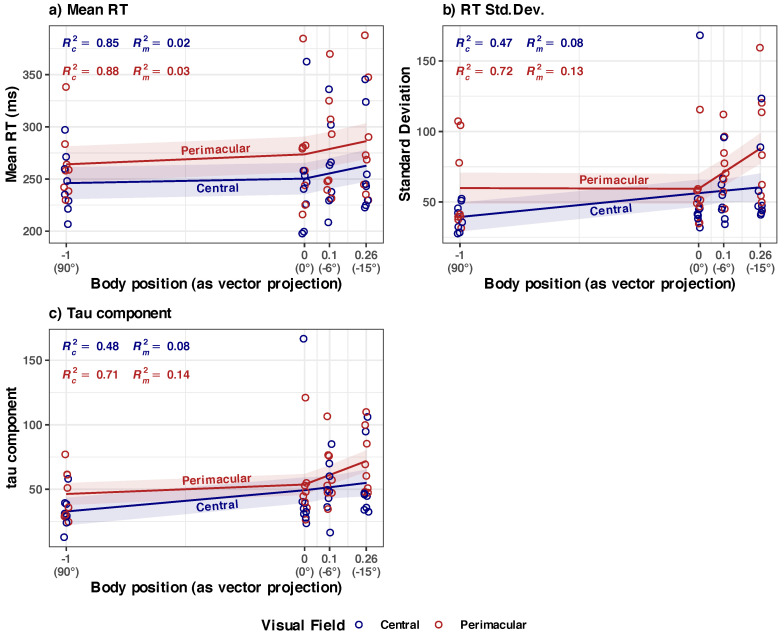
Body position effects on parameters of reaction times to visual stimuli presented in *central* vs. *perimacular* fields of view. LME modeling of (**a**) mean RT, (**b**) standard deviation of RT, and (**c**) ex-Gaussian fitting tau component of RT vs. body position. The body position is represented as vector projection values on the gravitational field, with the corresponding degrees of body position in parentheses. The LME models on the data are shown by a red line (perimacular visual field) and a blue line (central visual field); the HUT part of the models consists of the lines between 90° and 0°, and the HDT part are the lines between 0° and −15°. The bands are 95% CI intervals for the models. The circles represent the individual values from the participants, slightly automatically jittered on the horizontal axis to distinguish overlapping values. The numerical values are presented in Table A3.

The full details of the six models in Figure 8 are given in Appendix A.3 and Table A3, and a brief summary is given below:

**(a) Mean RT, in central vs. perimacular fields of view, at the group level** (Figure 8a)

*Summary*: The increase in Mean RTs appears to be different for the central and perimacular fields of view. There is an increase in mean RT from the central field of view in the HUT body position (90° to 0°), but it is not statistically significant. The main component of the increase happens in the perimacular field of view. The body position in HDT significantly (*p* = 0.015) influences the mean RT in the perimacular field of view, but the effect size is very small ($R_{m}^{2}$ = 0.03 in perimacular vs. 0.02 in central); only about a ~7.7% increase in the average RT occurred when the participants went from a vertical position (90°) to an inclined (−15°) position). In absolute terms, the perimacular mean RT time increased from 264.1 ms (at 90°) to 284.58 ms (at −15°). The perimacular–central mean RT difference was ~18 ms at 90° and slowly increased to 23.42 ms at −15°.

**Figure 9:**
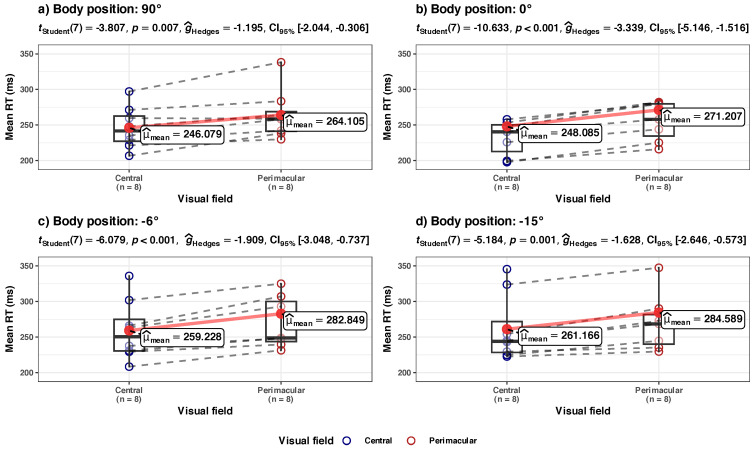
The differences between perimacular mean RT and central mean RT at the four body positions: (**a**) at 90°, (**b**) at 0°, (**c**) at −6°, and (**d**) at −15° tilt. The individual values are represented as circles; boxplots of the distributions are shown for each central/perimacular dataset. Red dots represent the sample mean ($\hat{\mu }$); a red line connects the dots as a guide to the eye. Student’s *t*-test values for each comparison are shown in captions of each panel. The effect size reported is Hedges’ $\hat{g}$. CI, confidence intervals.

To obtain an independent confirmation that there really is a separation between the models in central and perimacular areas, we analyzed the same data transversally by four separate Holm-adjusted pairwise *t*-tests performed between the values collected in the central vs. perimacular area, at each body position (see Figure 9). The differences in perimacular mean RT and central mean RT are statistically significant at each body inclination, with large effect sizes (the spread between the central and perimacular values seems to be consistent at each body position). Longitudinally, this would mean that there should be no overlap between the lines predicted by the two LME models, which seems to be the case in Figure 8a.

**(b) Std.Dev. RT in central vs. perimacular fields of viewat the group level** (Figure 8b)

*Summary*: The increase in the Std. Dev. of RTs appears to be different for central and perimacular fields of view. In the perimacular field, the body position in HDT significantly (*p* = 0.003) influences the increase in Std. Dev. of RTs; the model has a substantial power ($R_{c}^{2}$ = 0.72) and a larger influence on the fixed effect of the body position ($R_{m}^{2}$ = 0.13) than in the above presented models. However, for the central field, the model of Std. Dev. RT vs. body position appears to have only a medium explanatory power ($R_{c}^{2}$ = 0.47), and body position does not appear to have a significant influence.

**Figure 10:**
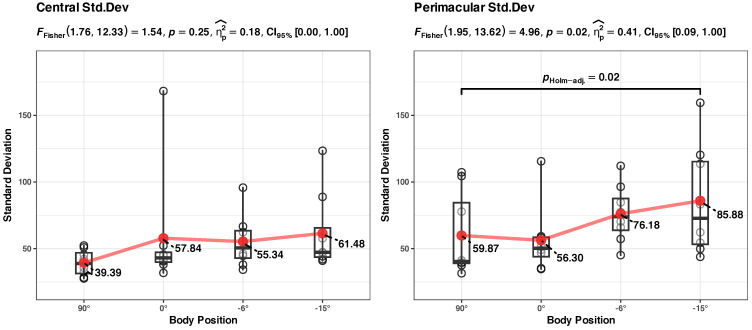
*Central* visual field (left panel) and *perimacular* visual field (right panel) repeated measures ANOVA of Std. Dev. RTs in the four body positions. The test results are reported in the subtitle of each panel. Gray dots: calculated standard deviation of RT values from each participant, overimposed on the boxplots of the data distribution. Red dots: the means at the group level. The red line is drawn as a guide to the eye. The bar shows significant post hoc inter-group comparisons (*t*-test); *p*-values reported are Holm-adjusted. $\hat{\eta_{p}^{2}}$, effect size for the sample comparisons (partial eta squared). CI, confidence intervals.

The LME modeling in Figure 8b indicates a sharp difference in the values of Std. Dev. RT in the perimacular area, between the body positions at 90° and −15°, while in the central area, the difference is much smaller. We independently checked the hypothesis of the differences between these states with a repeated measures ANOVA analysis (see Figure 10). This confirmed that, in the perimacular area, there is a significant difference between the groups, and the largest difference appears between the values at 90° and −15° (*p* = 0.02), with a large effect size ($\hat{\eta_{p}^{2}}$ = 0.41), while in the central area, differences in Std. Dev. RTs appear to be non-significant.

**(c) Tau component in central vs. perimacular fields of view at the group level** (Figure 8c)

*Summary:* The increase in tau component appears to be different for central vs. perimacular fields of view. In the perimacular field, the tau component increases from an average of 46.35 (at body position 90°) to 71.38 (at −15°); the body position in HDT significantly (*p* < 0.001) influences the increase. The perimacular tau LME model has a substantial power ($R_{c}^{2}$ = 0.71) and has the largest influence on the fixed effect of the body position ($R_{m}^{2}$ = 0.14) from all presented models. In contrast, in the central field of view, the increase is from an average of 32.8 to 55.10, and the body position does not appear to significantly influence the tau component.

**Figure 11:**
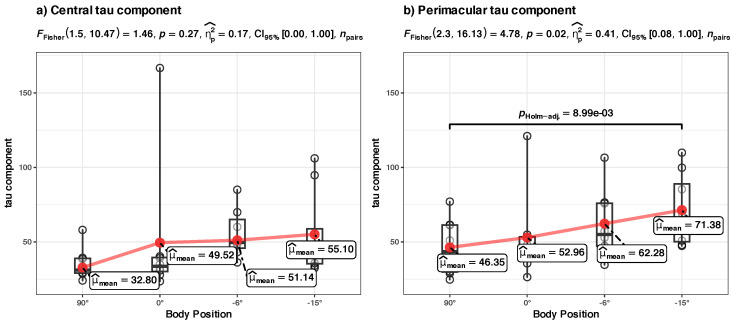
*Central* visual field (**a**) and *perimacular* visual field (**b**) repeated measures ANOVA of tau component in the four body positions. The test results are reported in the subtitle of each panel. Gray dots: calculated standard deviation of RT values from each participant, overimposed on the boxplots of the data distribution. Red dots: the means at the group level. The red line is drawn as a guide to the eye. The bar shows significant post hoc inter-group comparisons (*t*-test); *p*-values reported are Holm-adjusted. $\hat{\eta_{p}^{2}}$, effect size for the sample comparisons (partial eta squared). CI, confidence intervals.

We independently checked the hypothesis of the greater difference between the evolution of the tau component in the perimacular vs. central areas with repeated ANOVA analyses of the tau values in the two situations, grouped in the four body positions (see Figure 11). It confirmed that, in the perimacular area, there is a significant (*p* = 0.02) difference between the groups, and the largest difference appears between the values at 90° and −15° (*p* < 0.001) with a large effect size ($\hat{\eta_{p}^{2}}$ = 0.41), while in the central area, differences in tau component appear to be non-significant.

### 3.4. Visual-Motor Integration

*Paradigm: “Any kind of stimuli appearing in contralateral vs. ipsilateral visual fields*, *in different body positions*”.

*Hypothesis*: “*An acute exposure to a microgravity analogue would alter the distributions of the visual RTs in the contralateral vs. ipsilateral visual fields*”.

**Figure 12:**
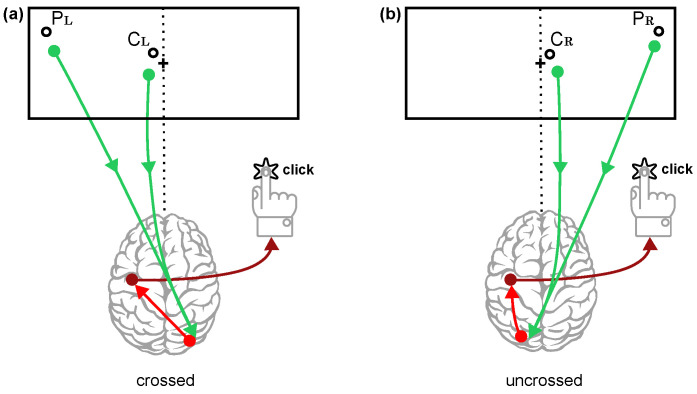
Crossed vs. uncrossed conditions. (**a**) Visual stimuli shown contralateral with the motor hand (“crossed” condition); (**b**) visual stimuli shown ipsilateral with the motor hand. $P_{L}$, $C_{L}$, $C_{R}$, and $P_{R}$ are the positions of the stimuli. Arrows: the simplified diagram of the information flow across hemispheric domains: green—visual information; brown—motor command; red—putatively hemispheric processing (central, frontal, and bilateral processing ignored in the diagram). Crossed—uncrossed difference (CUD) is the difference in RT parameters collected in condition (**a**) minus condition (**b**).

We carefully analyzed the impact of the subjects’ RTs on the lateralization of the stimuli. The stimuli were presented in either a crossed or uncrossed condition with the motor hand (see Figure 12). For each one of the investigated parameters of the RTs, we calculated the crossed–uncrossed difference (so-called CUD); we thus calculated CUD for the mean, median, μ, σ, τ, skewness, and kurtosis in each one of the four body positions. We then analyzed if there was any change in CUDs related to the body position from 90° to −15° (see Appendix B.5 and Figure A5 for an example of such analysis).

In our sample, we found no significant relationship between CUD and the change in the body position in the acute time frame we investigated. Thus, we cannot reject the null hypothesis of this paradigm, namely, that the acute exposure to microgravity does not appear to influence crossed–uncrossed difference of RTs.

### 3.5. High vs. Medium Visual Contrast

In our sample, we have found no significant difference between the investigated parameters of RTs to visual stimuli with medium contrast (50.58%) and those with high contrast (99.3%) in the four body positions.

**Figure A4:**
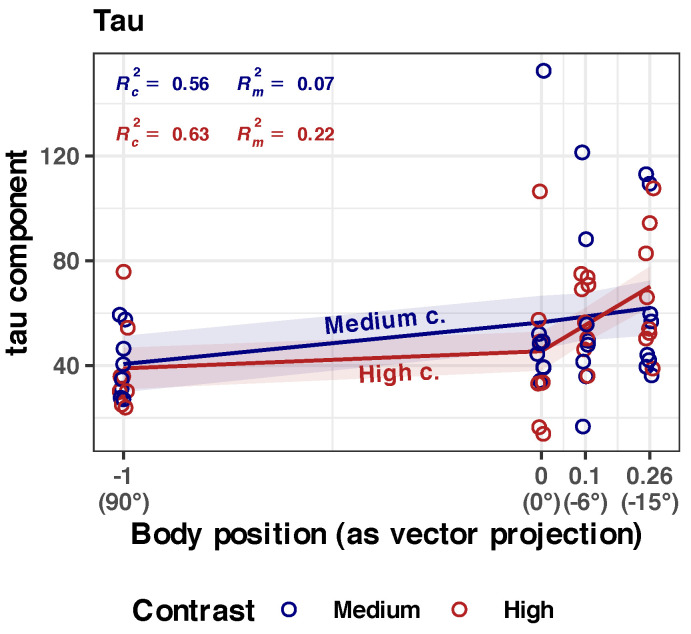
LME model of tau component of RTs to visual stimuli presented with medium contrast (50.58%) or high contrast (99.3%) vs. body position. Note: the model is non-significant, and lacks prediction power (the predicted values overlap—see the crossing at ~0.1), so its power to discriminate between the two conditions is very reduced. Additionally, there is also an almost complete overlap of CIs.

**Figure A5:**
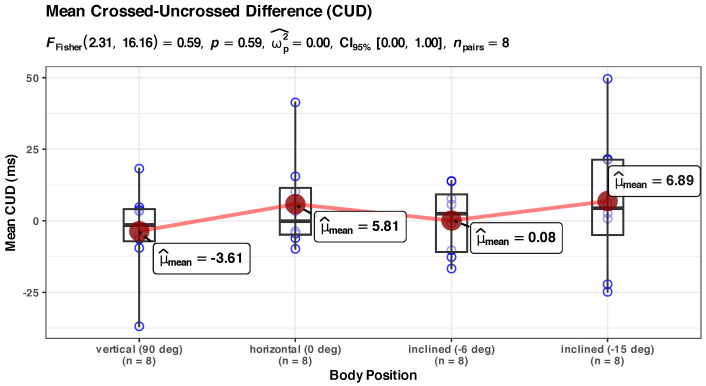
Normal CUD distribution range (no relationship with the body position found). Circles: individual CUD calculated values for each participant, overimposed on the boxplots showing the data distribution. Red dots, the sample mean in each position. The red line is drawn as a guide to the eye. Repeated measured ANOVA test, non-significant relationship (test results reported in the subtitle of the figure; the effect size, $\hat{\omega^{2}}$ indicates no effect in this sample).

As an example, at body position 90°, the difference between the means of all RTs of stimuli with medium contrast and those of stimuli with high contrast was 4.2 ms; at −15°, the difference of the means was 5.7 ms. The total change (from 90° to −15°) is thus 1.5 ms. This is within the limits of the temporal resolution of our setup (1 ms button switch), and we concluded that the variations we observed are the normal measurement fluctuations. A formal repeated measurements ANOVA of these differences vs. body position also did not show a significant statistical difference. (For the curious reader, in order to keep in line with the previous section, we present the LME model for the tau component for the contrasts of the stimuli as an example of a non-significant model; see Appendix B.4, Figure A4, and the comments in the *Discussion* section).

This was the design of our study (see *Methods*): we purposefully chose an intermediate contrast (medium contrast at 50.58%, well above the human detection threshold of ~0.5%) just as a means to add a small variation to the stimuli in order to prevent participants’ boredom in the repeated SRT task. We take the negative finding here as a confirmation that, for this setup, the easily recognizable visual contrasts of the stimuli we used posed no challenge to recognition or to response to our participants.

### 3.6. Other Anthropological Parameters

In our sample, we have found no other significant relationships between the changing parameters of RTs in different body positions and the anthropological parameters collected from our participants (age, sex, handedness, eye dominance).

## 4. Discussion

### 4.1. Quality of the Measurements

The human simple reaction time to visual stimuli is a broadly researched topic that shows that the distribution is typically right-tailed (positive skew) and it is characterized by large variability of response times, with reported means between 200 and 600 ms and with a standard deviation as high as 100 ms [34]. Our numerical results are within the expected range for human reaction times.

Similar distribution limits were previously reported in the literature: values smaller than 100 ms seem physiologically unreasonable (like 0.03 ms), but are found nevertheless (could be due to anticipation effects or by pure chance); uncharacteristically long RTs could be attributed to distraction, fatigue, or loss of attention (see [101] for a review). In order to avoid any bias, we analyzed the data as collected, with no exclusion of the extreme data points, as stated in the Methods section. The obtained fitted distributions (Figure 4 and Figure 5) are also in line with previously published research—a steep left slope and a gentler and wider right slope [40]. A particular property is the known synchronous relationship between the mean and the standard deviation of a response time distribution in most of the RT paradigms [102]. We have also observed this in our dataset.

### 4.2. Overall Group Results

#### Mean RT

A surprising result was the slight increase (~7%) in the overall mean reaction time in the first 10 min of HDT (so there is an overall *decrease* in performance in the simple task of visually detecting a stimulus and giving a motor response).

Previous studies reported seemingly conflicting results. It seems that the neuro-cognitive performance is enhanced during short periods (22 s) of microgravity induced by parabolic flights [30]; in their detailed analysis, Wollseiffen et al. [30] showed no change in RTs for simple tasks (in microgravity vs. normal gravity) but their task was a more complex one than SRT (involved recognition of a higher number out of two presented on the left and right sides of the visual field). For complex tasks (arithmetic problem solving), they found RTs were shorter in microgravity, but the task is very different from the SRT we employed. Other studies have shown decreases in RTs (improvement of performance) during an executive task after a longer HDT of 90 min [103]; these results were attributed to an increased oxygenation of the cortical areas during the microgravity (measured spectroscopically).

Opposite results were found by other groups. For instance, Dayal et al. [104] found a small decrease in speed (i.e., a small increase in RTs) with very small effect sizes after 4 h of HDT. Serial measurements performed every 2 hours in HDT (study by Komada et al. [105]) over several days found that, in a recognition task (a task a little more difficult than SRT), RTs were longer, but the difference was not statistically significant; on complex tasks (tracking tasks across a larger visual area), a decrease in performance (increased RTs) was significant. A recent study performed on the International Space Station (ISS) found a 39 ms increase in a RT task during spaceflight compared with that before and after flight [42].

As reconciling explanations, we propose the following:

- RTs are notoriously difficult to interpret, with many different models proposed; there is no model yet with a complete description of neural correlates of RTs [35,37,39,73,106];
- The experimental protocols are different; RTs are very sensitive to other confounding factors (discussed in *Methods*), and due to logistic and timing constraints on spaceflights, some of these factors were perhaps unaccounted for;
- The sample sizes are inconsistent, and there is a lack of standardization of the equipment used (with the associate timing errors discussed in *Methods*). All these factors diminish the signal-to-noise ratio of the measurements;
- A difficulty of comparison arises because of the different meanings of the term “acute” (referring to the time of exposure to microgravity). In the studies cited in this paper, we observed that the same “acute” term was given to very different time scales: seconds (in parabolic flight studies), minutes (in HDT), first 2 h, first 4 h or the first day (in HDT or immersion studies).

We interpret the visuo-motor RTs as a very time-sensitive property, and our results support the idea that the overall SRT time slightly increases following acute (10 min) exposure to a microgravity analogue.

We propose that at the center of the issue is the interplay between the following: (1) The complicated dynamics of the choroidal circulation [107]; (2) The very fast, body-wide response of the cardiovascular system to a microgravity stressor [51], (3) the autonomous nervous system response to stressors [108], and (4) a neuromechanical component [109].

Ocular changes in HDT are well documented [110]. During microgravity exposure, via a host of mechanisms [111], a blood pooling happens in the choroid and distends it [112], while the choroidal pulsatile blood flow is immediately reduced, which results in a net choroidal hypoperfusion [113], which can impair the function of the retinal rods and cones.

At the same time, the onset of microgravity is a cardiovascular stressor that, for a short time, activates the inhibitory component of the parasympathetic nervous system (PNS) [114], and it is known that parasympathetic stimulation causes choroidal vascular dilation and secondary increased blood flow [115] (thus further increasing the changes of the choroidal function). However, a subtle sampling problem appears with this PNS adaptive response. It seems that there are behaviorally measurable differences in PNS reactivity to stressors (different phenotypes in population) that lead to marked differences in inhibitory control to the same stressor; this is known to influence RTs [108]. Another different study linked performance-decreasing phenotypical variations (in metabolic pathways) to about 20% of the astronauts [10]. We think that these phenotypical variations might be an explanation for the high variability of the data collected in various HDT studies, and in our study as well.

The above considerations might explain why, in the immediate period following an acute microgravity stressor, a decrease in performance in SRT tasks might be observed, with a high degree of variability observed (from none to noticeably) depending on the individual factors of the participants. We think that this impairment is non-uniform also in the field of view due to a neuromechanical component that we present in the next subsection.

### 4.3. Central vs. Perimacular Field of View

It is known that SRT increases as a function of the eccentricity in the visual field [65,116,117]. We have observed that the increased mean RT (and also Std. Dev. and τ) is consistently higher in the perimacular field of view rather than in the central one (as expected) in the vertical situation (Figure 8a and Figure 9a).

One of our novel findings is the observation that the exposure to microgravity seems to affect the perimacular area more severely than the central area (red lines in Figure 8b,c). Additionally, in the perimacular area, there seems to be a disconnect between the normal linear link of the mean RT and Std. Dev. RT [102] (red lines in Figure 8a,b vs. blue lines in the same figure). This indicates different dynamics for the perimacular area vs. central area when exposed to HDT.

As a possible explanation for our results, we propose that the retinal tissue is affected by the microgravity (HDT in this case) in a gradient-wise fashion. It is known that other tissues of the body are affected by exposure to microgravity in a non-uniform way. For instance, the muscle tissue deconditioning in microgravity has different proximal-to-distal rates [118], cardiac sphericity ratio changes in microgravity [119], and non-uniform bone density changes [120,121]. We propose that similar differential changes occur in the retina.

One of the hallmarks of the exposure to microgravity is an increased fluid translocation from the vascular lumen to the interstitial space, particularly in the upper body tissues [122], which is sometimes so large that even results in noticeable macroscopical enlargement of the tissues (“puffy face” or “stuffed nose”) of the astronauts, as a result of the many adaptive cardiovascular changes to microgravity [122]. It is known that the choroidal blood volume doubles within seconds from exposure to microgravity [123], and that enlargement of the choroidal tissue is measurable within 15 min of exposure to microgravity [112].

**Figure 13:**
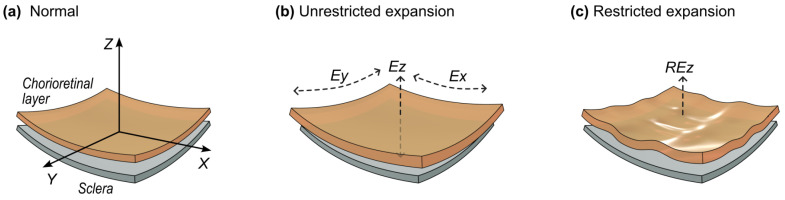
A diagram of a small patch of the eye wall depicting the curvature of the discussed layers (see text), as seen from the inside of the eye. Orange: the inner layer (chorioretinal); gray: the outer layer (sclera). The frame of reference is local to the patch, XY coordinates referring to the tangential surface of the patch, and the local vertical *Z* axis pointing towards the center of the eye. (**a**) The normal situation in healthy state: the two layers are parallel to each other and have roughly the same area. (**b**) In the case of a hypothetically unrestricted expansion of the inner layer (chorioretinal), the expansion components (Ex, Ey, Ez) are each one isotropic on its axis. Ex and Ey are increasing the area and Ez manifests as thickening. (**c**) An area-restricted expansion; the constraint is the inner surface of the eye (total surface of the sclera is constant). The folds form to accommodate the additional area of the expanded layer on the same area of the constant layer below it; thus an uneven expansion on the *Z* axis appears (REz). In order to aid the visualization, a small gap is shown between the sclera and chorioretinal layer; in all three panels, the gap has exactly the same size. The reference patch (the sclera) has identical dimensions in all panels. Additionally, see the Supplemental Materials Videos S1 and S2 for animations of (**b**,**c**).

We speculate that this fluid translocation alters the geometrical micro-arrangements of the eye tissues. We think that there are different dynamics of the two adjacent layers: sclera (external) and chorioretinal layer (internal) (see Figure 13a). The sclera, being more fibrous and less vascularized, is less influenced by the fluid translocation [124]; it is thus the limiting geometric factor. On the other hand, the chorioretinal layer, with an important microvascular component, is more influenced by the microgravity.

In prolonged microgravity, the fluid translocation from the intravascular to the interstitial space would enlarge the tissue as a whole in all directions, if there would be no constraints on this enlargement. On the local normal *Z*-axis, this would be perceived as thickening, but in the local XY axes, this would integrate as an increase in the total choroidal area (Figure 13b). However, as a whole, the choroidal tissue is spherically shaped and an increase in the area would mean an increase in its radius, but it is geometrically constrained by the spherical sclera adjacent to it. Therefore, the gained area would have to fold within (see Figure 13c and the Supplemental Materials Videos S1 and S2). Thus, anatomically, in time, chorioretinal folds would form (a hallmark of SANS); of course, this mechanical cause would be further complicated and modified by the internal cellular response to mechanical stress [125].

#### 4.3.1. Mechanical Stress Dynamics in Time

Functionally, this folding process is a mechanical stress on the tissue (choroid and retina). The mechanical stress precedes folding. It is known that local mechanical stress plays a significant role in neuronal function [125], and excess mechanical force and deformation lead to structural changes and functional impairment [126]; mechanical stretching and force play a significant role in modulating the structure and function of the nervous tissue (neuromechanics effects) [109]. The retinal neurons have a fast response (mediated by internal ${\mathrm{Ca}}^{2+}$ions) to microenvironment pressure elevation, which leads to functional and morphological changes [127]. It is known from animal model studies that the electrical activity of the retinal ganglion cells is impaired immediately after an elevation of the intraocular pressure (lab controlled, mechanically induced) and that it promptly normalizes after the cessation of the mechanical stressor [128]. Human studies have shown that HDT diminishes the electrical activity at retinal levels within 1 min of the exposure [27].

Thus, we propose that the intra-tissular mechanical stress in the genesis of the chorioretinal folding process can potentially induce functional changes in the retina (via the neuromechanic effects briefly mentioned above). This proposed explanation would hold regardless of the exact manner by which the mechanical stress of the HDT affects the ocular globe (translaminar gradient, cephalad fluid shifts, venous return impairment, jugular distention, decreased choroidal venous drainage, and secondary stagnation or pooling of blood in the choroid etc. [1,4,14,26,27,122,129]).

Consequently, at the onset of the mechanical stress from the outside of the eye globe, the mechanical stress itself would also alter the function of the cells within. Further research is needed to quantitatively link the mechanical stress component with the functional changes at the cellular level. We think that this functional impairment is very mild (not detectable by standard vision tests, but by specific functional tests) and might be a possible explanation for our observed increase in mean RT and decrease in RTs’ accuracy (increased RT Std. Dev. and τ component) immediately following HDT.

#### 4.3.2. The Distribution of This Mechanical Stress in the Chorioretinal Layer as a Whole

We hypothesize that this mechanical stress would be nonlinearly distributed in the chorioretinal layer, with a larger component of the stress in the folding and shearing stress zones. The rigorous study of tissue folding in general and its numerical modeling is a relatively new field (see [130] for a review). The analysis of the folding of multiple layered models is particularly difficult, but numerical models of the situations where a soft tissue folds on another layer with a different elastic properties were performed [131], with results that predict pattern shapes in brain cortical folds, intestinal villi, etc. It seems that if there is a variable layer-to-substrate modulus ratio (i.e., one tissue’s elasticity and stiffness properties varies along the tissue), an anisotropic stress is produced within the growing soft tissue, which can lead to complex sulcification patterns [131].

We argue that this might be the case with the scleral–chorioretinal coupling (tighter coupling towards the optic disc due to anisotropy in choroidal vasculature [107] and to the presence of the optic head—a large anisotropic element). We think that these high shearing stress zones are therefore distributed adjacently to the optical disc and the lower stress zones are farther away. This would (presumably) lead to folding stress patterns extending radially from the optic disc and gradually diminishing. Therefore, the neuromechanical stress would be higher in the peripapillary area (located peripherally in the field of vision); this would lead to a greater functional impairment of the neurons in this area rather than in the foveal area. This would probably explain our results that show a greater impairment of function in the perimacular area (particularly towards the circumpapillary area) compared with the foveal area (greater mean RT, Std. Dev., τ). While the exact genesis of the chorioretinal folds is unknown, spatial patterns of folding like this are reported [132], which underscores the importance of the differential investigation of the central vision and the more peripheral retina. Our results support the observation of Ferguson et al. [132] that the choroidal folds develop primarily in the superior, nasal, and inferior areas to the optic nerve head (we sampled the supero-nasal area in $P_{L}$ and $P_{R}$, Figure 2a).

The above hypotheses might explain our results of different effects of HDT on RT distribution on the central vs. perimacular portions of the retina. Whether the PNS inhibitory activity (discussed above) has a different role in perimacular vs. central retina in HDT is currently unknown.

Different complementary (and not necessarily exclusive) explanations would be related to the retinal topology changes with eccentricity, the most important being the different distributions of the cones and rods and the different types of neurons: magno- and parvo-cellular (M and P) pathways. Preliminary studies suggest that M and P pathways appear to have slightly different metabolic requirements [133]; whether these might be incongruously influenced by microgravity stress is still an open question.

### 4.4. Visual-Motor Integration

The crossed–uncrossed difference (CUD) is generally taken to be a rough estimate of the interhemispheric transfer time and the normal range lies within 2–6 ms [134]. Typically, reaction times are faster for uncrossed responses (the stimulus and the motor hand on opposite sides), with complex cerebral dynamics [135,136]. Modifications of peripheral RT and evaluation of CUD seem to be sensitive enough to detect mild traumatic brain injury [137].

Our results are within the normal variation range, −3.61 ms to 6.89 ms (for a sample of around 200 RTs recorded, a number of negative CUD findings are statistically expected in the population [138]).

The fact that we did not find a CUD change implies that acute exposure to microgravity analogues does not significantly alter the brain processing.

Our results might be seen as an indirect confirmation of Wollseiffen et al. [30], who suggested that cortical processes are not impaired but actually improved in microgravity. Therefore, we think that our above-discussed results (RTs increase) are mainly attributable to the performance degradation within the ocular structures. Even if the CUD result is a negative finding, we note that it is known that high statistical power in a CUD study would be yielded by thousands of repetitions of the task [138], which cannot be performed in a time-limited microgravity study).

### 4.5. High vs. Medium Visual Contrast

In this study, we did not investigate the contrast sensitivity as a function of retinal eccentricity in microgravity settings.

By design, we wanted to avoid the influence of the contrast on RTs, as it is known that RTs are heavily influenced by the luminance and contrast [74,117]. We strove for the conditions for the best RTs: in the settings of a luminous white background and large stimulus, the visual performance reaches an asymptotic level [139]. Therefore, the negative findings we report seem to be a confirmation of the research methodology we employed.

However, in line with the above-presented results, we would like to highlight the importance of a contrast sensitivity study in the peripheral retina in microgravity studies [140], even if it is technically challenging to perform accurately. If the functional impairment reported by this study is transferable to the contrast sensitivity studies, we suppose that the contrast threshold would be higher in the peripheral retina in microgravity than under normal conditions; further research is needed.

### 4.6. Implications

Our results provide an independent additional support for the idea that vision assessment of astronauts during spaceflight should probably be expanded [141]. The classical Amsler grid vision test, which is used also on the ISS, covers an eccentricity of about 10° from the foveal fixation. Our results detected performance changes in an acute microgravity analogue in more eccentrical retina (~20°), which is not covered by the Amsler grid or by the static visual acuity tests. The implication would be that functional testing of the perimacular retina might be beneficial for the earlier detection of SANS-related ailments in addition to the regular testing of the central vision.

#### Limitations

We acknowledge the limitations of this pilot exploratory study. The main limitation is related to the limited sample size (*n* = 8). While our findings might not extrapolate to all participants or cases, for these kinds of studies, other exploratory studies used a similar number of participants (see for instance [12,105]). Additionally, our sample is not representative for all age groups (our participants were healthy young adults); the results should be not generalized until confirmation by larger follow-up studies. We cannot rule out spurious factors outside of our control, for instance, the individual stress levels of the participants. It is known from previous studies that decreased performance can be related to increased stress levels rather than to microgravity [30]. We attempted to diminish the effect of stress by our time-relaxed protocol spread over several weeks, with a guided tour of the lab, a demonstration of the measurements (see Methods). We always asked the participants if they were comfortable before each measurement, but we cannot exclude situations of misreported stress levels or performance ability [142,143].

## 5. Conclusions

The results of our exploratory study suggest that the SRT distribution shape changes subtly but significantly during acute exposure to microgravity analogues. The effect sizes are larger in the perimacular retina, and the central retina seems to be largely unaffected by the acute exposure to microgravity. Our findings might explain the elusiveness of SANS diagnosis in the acute phase—usual tests for central vision (visual acuity, contrasts) are less likely to detect the early pathological changes in the retina in SANS. The data obtained in our sample suggest that the diagnostic tests should more thoroughly investigate the extra-foveal areas, notably the perimacular area towards the optic nerve head.

These findings may have important implications regarding early diagnosis of SANS, namely, including additional close monitoring of visual parameters in extra-foveal areas (and particularly perimacular areas, peri-blind spot areas), which are usually not tested in routine visual exams (for instance, the Amsler grid test covers an eccentricity of about 10°, while the reported results are ~20° eccentricity).

## Figures and Tables

**Figure A2:**
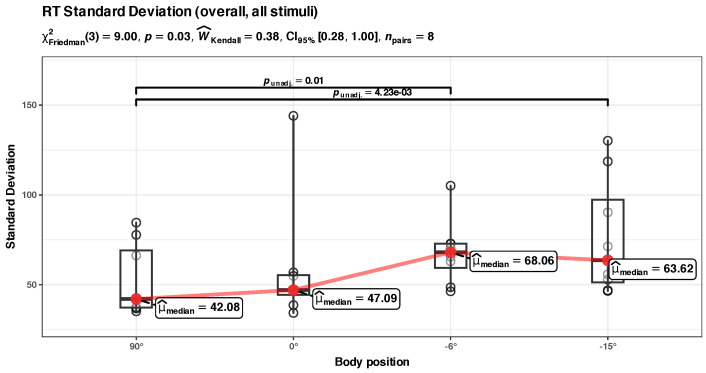
A p-unadjusted group analys (Friedman rank sum test) of the LME model in Figure 6b and Figure 7. Gray dots: calculated standard deviation of RT values from each participant, overimposed on the boxplots of the data distribution. Red dots: the medians at the group level. The red line is drawn as a guide to the eye. Inter-group unadjusted comparisons indicate that there is a resizable difference also between 90° (vertical) and −6°, not only between 90° and −15° (as shown in an adjusted analysis, Figure 7). This upward trend is picked up by the LME model, as shown in Figure 6b.

## Data Availability

Data gathered by the experimental protocol described above is available on the Zenodo public data repository, https://zenodo.org/doi/10.5281/zenodo.11840653 (accessed on 16 June 2024).

## Supplementary Materials

The following are available online at https://www.mdpi.com/article/10.3390/diagnostics14171918/s1, Video S1: Unrestricted_expansion.webm, Video S2: Restricted_expansion.webm.

## Abbreviations

The following abbreviations are used in this manuscript:

ANOVA	analysis of variance
CI	confidence interval
DC	direct current
HDMI	High-Definition Multimedia Interface
HDT	head-down tilt
HUT	head-up tilt
ID	the unique identifier of a participant’s responses in the dataset
IPS	in-plane switching
ISS	International Space Station
LCD	liquid-crystal display
LED	light-emitting diode
LME	linear mixed effects
PNS	parasympathetic nervous system
SANS	Spaceflight-Associated Neuro-Ocular Syndrome
SRT	simple reaction time
Std. beta	standardized beta
Std. Dev.	standard deviation
RT	reaction time
OCT	optical coherence tomography

## Appendix A. Statistical Details

For completeness, in Appendix A, we list the numerical equivalents of the models and graphics in the article.

### Appendix A.1. Descriptive Group Statistics

**Table A1:** The group statistics of the measured reaction times (in milliseconds).

Characteristic	Body Position			
	Vertical (90°), *N* = 8	Horizontal (0°), *N* = 8	Inclined (−6°), *N* = 8	Inclined (−15°), *N* = 8
Median				
(ms)	246.84	245.64	255.72	255.55
Mean				
(ms)	255.09	259.64	271.04	272.88
SD	52.79	58.68	68.14	76.59
Skewness	1.93	1.83	2.54	2.64
Kurtosis	11.16	7.52	11.27	16.46
mu	215.29	211.03	212.98	212.81
sigma	21.86	22.96	19.48	22.52
tau	40.07	48.46	58.13	60.22
logLIK	−578.69	−582.53	−595.76	−613.66
AIC	1163.38	1171.05	1197.52	1233.31
BIC	1171.54	1179.21	1205.68	1241.47

SD: standard deviation of the mean; logLIK: log likelihood; AIC: Akaike information criterion; BIC: Bayesian information criterion; LogLIK, AIC, and BIC values for the ex-Gaussian fitting yielding mu, sigma, tau values.

### Appendix A.2. Overall Group Models

**Table A2:** Characteristics of the linear mixed-effects (LME) models for overall group results, presented in Figure 6.

	Dependent Variable:		
	Mean RT	RT St.Dev.	tau
$\beta_{1}$	6.91	6.75	10.31
	(7.72)	(7.36)	(6.78)
$\beta_{2}$	19.40 *	24.39 **	21.46 **
	(7.99)	(7.61)	(7.01)
Constant	255.09 ***	52.79 ***	40.07 ***
	(16.17)	(9.75)	(8.38)
Observations	32	32	32
Conditional $R^{2}$	0.87	0.71	0.67
Marginal $R^{2}$	0.02	0.10	0.10
Log Likelihood	−137.32	−132.10	−129.07
Akaike Inf. Crit.	284.64	274.20	268.14
Bayesian Inf. Crit.	291.97	281.53	275.47

Notes: * *p* < 0.05; ** *p* < 0.01; *** *p* < 0.001; the numbers in parentheses are the standard errors. $\beta_{1}$—slope for HUT, body position modeled as vector projection when body inclination is [90°…0°) above the horizontal plane; $\beta_{2}$—slope for HDT, body position modeled as vector projection when body inclination is [0°…−15°] below the horizontal plane.

**(a) LME model for mean RT at the group level**: Figure 6a

R formula: *Mean ~ bs(BodyPosition, knots = c(0), degree = 1) + (1|ID)*. The model included ID as random effect (formula: ~1|ID). The model’s total explanatory power is substantial (conditional $R^{2}$ = 0.87), and the part related to the fixed effects alone (marginal $R^{2}$) is 0.02. The model’s intercept, corresponding to Body Position = 0, is at 255.09 (95% CI [221.91, 288.28], t(27) = 15.77, *p* < 0.001). Within this model are as follows:

- The effect of Body Position [HUT] is statistically non-significant and positive ($\beta_{1}$ = 6.91, 95% CI [−8.94, 22.76], t(27) = 0.89, *p* = 0.379; Std. beta = −0.02, 95% CI [−0.53, 0.50]).
- The effect of Body Position [HDT] is statistically significant and positive ($\beta_{2}$ = 19.40, 95% CI [3.00, 35.79], t(27) = 2.43, *p* = 0.022; Std. beta = 0.44, 95% CI [0.07, 0.81]).

**(b) LME model for RT standard deviation at the group level**: Figure 6b

R Formula: *StDev ~ bs(BodyPosition, knots = c(0), degree = 1) + (1|ID)*. The model included ID as random effect (formula: ~1|ID). The model’s total explanatory power is substantial (conditional R^2^ = 0.71), and the part related to the fixed effects alone (marginal R^2^) is 0.10. The model’s intercept, corresponding to Body Position = 0 (at 0° tilt), is at 52.79 (95% CI [32.78, 72.79], t(27) = 5.41, *p* < 0.001). Within this model are as follows:

- The effect of Body Position [HUT] is statistically non-significant and positive ($\beta_{1}$ = 6.75, 95% CI [−8.35, 21.85], t(27) = 0.92, *p* = 0.367; Std. beta = −0.15, 95% CI [−0.92, 0.63]).
- The effect of Body Position [HDT] is statistically significant and positive ($\beta_{2}$ = 24.39, 95% CI [8.77, 40.01], t(27) = 3.20, *p* = 0.003; Std. beta = 0.87, 95% CI [0.31, 1.43]).

**(c) Tau parameter at the group level** (Figure 6c)

R formula: *tau ~ bs(BodyPosition, knots = c(0), degree = 1) + (1|ID)*. The model included ID as random effect (formula: ~1|ID). The model’s total explanatory power is substantial (conditional $R^{2}$ = 0.67), and the part related to the fixed effects alone (marginal $R^{2}$) is 0.10. The model’s intercept, corresponding to Body Position = 0, is at 40.07 (95% CI [22.87, 57.28], t(27) = 4.78, *p* < 0.001). Within this model are the following:

- The effect of BodyPosition [HUT] is statistically non-significant and positive ($\beta_{1}$ = 10.31, 95% CI [−3.60, 24.22], t(27) = 1.52, *p* = 0.140; Std. beta = 0.14, 95% CI [−0.69, 0.98]).
- The effect of Body Position [HDT] is statistically significant and positive ($\beta_{2}$ = 21.46, 95% CI [7.07, 35.85], t(27) = 3.06, *p* = 0.005; Std. beta = 0.89, 95% CI [0.29, 1.49]).

### Appendix A.3. Central vs. Perimacular Fields of View Models

**Table A3:** Characteristics of the linear mixed-effects models of the body position effects on central vs. perimacular field of view, presented in Figure 8.

	Dependent Variable:					
	Mean RT		RT Std. Dev.		tau	
	Central	Peri.	Central	Peri.	Central	Peri.
$\beta_{1}$	4.29	9.54	16.90	−0.47	16.47	7.34
	(7.97)	(8.20)	(10.24)	(8.31)	(10.59)	(6.50)
$\beta_{2}$	16.65	22.14 *	21.02	28.12 **	22.13	25.52 ***
	(8.24)	(8.48)	(10.59)	(8.60)	(10.96)	(6.73)
Constant	246.08 ***	264.11 ***	39.39 ***	59.87 ***	32.80 **	46.35 ***
	(15.31)	(17.21)	(10.20)	(10.95)	(10.66)	(8.48)
Obs.	32	32	32	32	32	32
Cond. $R^{2}$	0.85	0.88	0.47	0.72	0.48	0.71
Margin. $R^{2}$	0.02	0.03	0.08	0.13	0.08	0.14
LogLIK	−137.54	−139.07	−138.70	−135.57	−139.81	−128.37
AIC	285.09	288.15	287.40	281.13	289.61	266.75
BIC	292.42	295.48	294.72	288.46	296.94	274.07

Notes: * *p* < 0.05; ** *p* < 0.01; *** *p* < 0.001; the numbers in parentheses are the standard errors; Peri.—perimacular; $\beta_{1}$—slope for HUT, body position modeled as vector projection when body inclination is [90°…0°) above the horizontal plane; $\beta_{2}$—slope for HDT, body position modeled as vector projection when body inclination is [0°…−15°] below the horizontal plane.

**(a) Mean RT** (Figure 8a, Table A3)

**Mean RT in central field of view at the group level**

The LME model R formula: *Mean ~ bs(BodyPosition, knots = c(0), degree = 1) + (*1|ID*)*; the model included ID as random effect (formula: ~1|ID), within the data collected from the central field of view. The model’s total explanatory power is substantial (conditional $R^{2}$ = 0.85), and the part related to the fixed effects alone (marginal $R^{2}$) is 0.02. The model’s intercept, corresponding to Body Position = 0 (at 0° tilt), is at 246.08 (95% CI [214.66, 277.50], t(27) = 16.07, *p* < 0.001). Within this model are as follows:

- The effect of Body Position [HUT] is statistically non-significant and positive ($\beta_{1}$= 4.29, 95% CI [−12.05, 20.64], t(27). = 0.54, *p* = 0.594; Std. beta = −0.08, 95% CI [−0.64, 0.48])
- The effect of Body Position [HDT] is statistically non-significant and positive ($\beta_{2}$ = 16.65, 95% CI [−0.26, 33.56], t(27) = 2.02, *p* = 0.053; Std. beta = 0.40, 95% CI [−0.0063, 0.80]).

**Mean RT in perimacular field of view at the group level**

The LME model R formula: *Mean ~ bs(BodyPosition, knots = c(0), degree = 1) + (1|ID)*; incuding ID as random effect (formula: ~1|ID), within the data collected from the perimacular field of view. The model’s total explanatory power is substantial (conditional $R^{2}$ = 0.88), and the part related to the fixed effects alone (marginal $R^{2}$) is 0.03. The model’s intercept, corresponding to Body Position = 0 (at 0° tilt), is at 264.11 (95% CI [228.79, 299.42], t(27) = 15.34, *p* < 0.001). Within this model are as follows:

- The effect of Body Position [HUT] is statistically non-significant and positive ($\beta_{1}$ = 9.54, 95% CI [−7.29, 26.36], t(27) = 1.16, *p* = 0.255; Std. beta = 0.04, 95% CI [−0.48, 0.55]).
- The effect of Body Position [HDT] is statistically significant and positive ($\beta_{2}$ = 22.14, 95% CI [4.74, 39.55], t(27) = 2.61, *p* = 0.015; Std. beta = 0.47, 95% CI [0.10, 0.84]).

**(b) Std. Dev. RT** (Figure 8b, Table A3)

**Std. Dev. in central field of view at the group level**

The LME model R formula: *StDev ~ bs(BodyPosition, knots = c(0), degree = 1) + (1|ID)*; the model included ID as random effect (formula: ~1|ID), within the data collected from the central field of view. The model’s total explanatory power is medium (conditional $R^{2}$ = 0.47), and the part related to the fixed effects alone (marginal $R^{2}$) is 0.08. The model’s intercept, corresponding to Body Position = 0 (at 0° tilt), is at 39.39 (95% CI [18.46, 60.31], t(27) = 3.86, *p* < 0.001). Within this model are as follows:

- The effect of BodyPosition [HUT] is statistically non-significant and positive ($\beta_{1}$ = 16.90, 95% CI [−4.11, 37.91], t(27) = 1.65, *p* = 0.111; Std. beta = 0.50, 95% CI [−0.55, 1.54]).
- The effect of Body Position [HDT] is statistically non-significant and positive ($\beta_{2}$ = 21.02, 95% CI [−0.71, 42.76], t(27) = 1.98, *p* = 0.057; Std. beta = 0.73, 95% CI [−0.02, 1.48]).

**Std. Dev. in perimacular field of view** **at the group level**

The LME model R formula: *StDev ~ bs(BodyPosition, knots = c(0), degree = 1) + (1|ID)*; including ID as random effect (formula: ~1|ID), within the data collected from the perimacular field of view. The model’s total explanatory power is substantial (conditional $R^{2}$ = 0.72), and the part related to the fixed effects alone (marginal $R^{2}$) is 0.13. The model’s intercept, corresponding to Body Position = 0 (at 0° tilt), is at 59.87 (95% CI [37.41, 82.32], t(27) = 5.47, *p* < 0.001). Within this model are as follows:

- The effect of Body Position [in HUT] is statistically non-significant and negative ($\beta_{1}$ = −0.47, 95% CI [−17.52, 16.57], t(27) = −0.06, *p* = 0.955; Std. beta = −0.57, 95% CI [−1.33, 0.20]).
- The effect of Body Position [in HDT] is statistically significant and positive ($\beta_{2}$ = 28.12, 95% CI [10.48, 45.76], t(27) = 3.27, *p* = 0.003; Std. beta = 0.88, 95% CI [0.33, 1.44])
  .

**(c) Tau component** (Figure 8c, Table A3)

**Tau component in central field of view at the group level**

The LME model R formula: *tau ~ bs(BodyPosition, knots = c(0), degree = 1) + (*1|ID*)*; the model included ID as random effect (formula: ~1|ID), within the data collected from the central field of view. The model’s total explanatory power is substantial (conditional $R^{2}$ = 0.48), and the part related to the fixed effects alone (marginal $R^{2}$) is 0.08. The model’s intercept, corresponding to Body Position = 0 (at 0° tilt), is at 32.80 (95% CI [10.93, 54.67], t(27) = 3.08, *p* = 0.005). Within this model are as follows:

- The effect of Body Position [HUT] is statistically non-significant and positive ($\beta_{1}$ = 16.47, 95% CI [−5.27, 38.21], t(27) = 1.55, *p* = 0.132; Std. beta = 0.43, 95% CI [−0.60, 1.46]).
- The effect of Body Position [HDT] is statistically non-significant and positive ($\beta_{2}$ = 22.13, 95% CI [−0.36, 44.62], t(27) = 2.02, *p* = 0.054; Std. beta = 0.73, 95% CI [−0.01, 1.48]).

**Tau component in perimacular field of view at the group level**

The LME model R formula: *tau ~ bs(BodyPosition, knots = c(0), degree = 1) + (1|ID)*; The model included ID as random effect (formula: ~1|ID). The model’s total explanatory power is substantial (conditional $R^{2}$ = 0.71), and the part related to the fixed effects alone (marginal $R^{2}$) is 0.14. The model’s intercept, corresponding to Body Position = 0 (at 0° tilt), is at 46.35 (95% CI [28.95, 63.75], t(27) = 5.46, *p* < 0.001). Within this model are as follows:

- The effect of Body Position [HUT] is statistically non-significant and positive ($\beta_{1}$ = 7.34, 95% CI [−6.01, 20.68], t(27) = 1.13, *p* = 0.269; Std. beta = −0.15, 95% CI [−0.93, 0.62]).
- The effect of Body Position [HDT] is statistically significant and positive ($\beta_{2}$ = 25.52, 95% CI [11.72, 39.33], t(27) = 3.79, *p* < 0.001; Std. beta = 1.03, 95% CI [0.47, 1.58]).

## Appendix B. Miscellaneous Low-Significance Findings

In this article, we reported findings that met the stringent statistical criteria for significance and effect size (see Methods). However, this being a pilot study, in this Appendix B, we list some exploratory analyses performed without Bonferroni–Holm correction, as suggested for exploratory studies by [89,90,91,144].
